# Small GTPase Cdc42, WASP, and scaffold proteins for higher-order assembly of the F-BAR domain protein

**DOI:** 10.1126/sciadv.adf5143

**Published:** 2023-04-26

**Authors:** Wan Nurul Izzati Wan Mohamad Noor, Nhung Thi Hong Nguyen, Theng Ho Cheong, Min Fey Chek, Toshio Hakoshima, Takehiko Inaba, Kyoko Hanawa-Suetsugu, Tamako Nishimura, Shiro Suetsugu

**Affiliations:** ^1^ Division of Biological Science, Graduate school of Science and Technology, Nara Institute of Science and Technology, 8916-5, Takayama, Ikoma, Nara 630-0192, Japan.; ^2^ Data Science Center, Nara Institute of Science and Technology, 8916-5, Takayama, Ikoma, Nara 630-0192, Japan.; ^3^ Center for Digital Green-Innovation, Nara Institute of Science and Technology, 8916-5, Takayama, Ikoma, Nara 630-0192, Japan.

## Abstract

The higher-order assembly of Bin-amphiphysin-Rvs (BAR) domain proteins, including the FCH-BAR (F-BAR) domain proteins, into lattice on the membrane is essential for the formation of subcellular structures. However, the regulation of their ordered assembly has not been elucidated. Here, we show that the higher ordered assembly of growth-arrested specific 7 (GAS7), an F-BAR domain protein, is regulated by the multivalent scaffold proteins of Wiskott-Aldrich syndrome protein (WASP)/neural WASP, that commonly binds to the BAR domain superfamily proteins, together with WISH, Nck, the activated small guanosine triphosphatase Cdc42, and a membrane-anchored phagocytic receptor. The assembly kinetics by fluorescence resonance energy transfer monitoring indicated that the GAS7 assembly on liposomes started within seconds and was further increased by the presence of these proteins. The regulated GAS7 assembly was abolished by Wiskott-Aldrich syndrome mutations both in vitro and in cellular phagocytosis. Therefore, Cdc42 and the scaffold proteins that commonly bind to the BAR domain superfamily proteins promoted GAS7 assembly.

## Teaser

A small GTPase and active receptor promote the higher-order assembly of the F-BAR domain protein with multivalent scaffold proteins.

## INTRODUCTION

The shape of the cellular membrane is mainly determined by the protein assemblies beneath the membrane. The Bin-amphiphysin-Rvs (BAR) domain superfamily proteins, including the FCH-BAR (F-BAR) domain protein family, assemble into the highly ordered oligomers of lattice-like assembly to shape and sense the membrane curvature and generate a platform of proteins (*1*, *2*). The oligomer surface of the BAR domains is thought to correspond to the shapes of the membranes required for their activities (*3*–*5*). However, the mechanism by which the BAR domains oligomerize downstream of the intracellular signaling cascade has remained enigmatic.

The membrane binding of BAR domain proteins has been analyzed by various methodologies, including liposome sedimentation (*6*–*8*). However, the extent of the oligomeric assembly of the BAR domain proteins has been less explored, with the exceptions of electron microscopy studies and superresolution analyses (*5*, *9*), which have longer time resolutions. The difficulty in analyzing the oligomeric assembly of BAR domain proteins presumably results from the relatively small number of oligomeric BAR domains on narrow membrane tubules of several hundred nanometers. On the other hand, growth-arrested specific 7 (GAS7) is involved in the formation of the phagocytotic cup (*9*), a micrometer-sized membrane domain. Therefore, the numbers of GAS7 proteins in the oligomeric clusters were expected to be larger than those in other BAR domain–containing protein oligomers, because the membrane structures are micrometer-sized.

Most BAR domain–containing proteins have Src homology 3 (SH3) domains and/or WW domains that interact with the proline-rich region (PRR) of Wiskott-Aldrich syndrome protein (WASP) family (*10*), of which WASP and its ubiquitous isoform, neural WASP (N-WASP), are similar in amino acid sequence each other. The BAR domain itself self-oligomerizes. However, the role of SH3 domain binding to PRRs and their multivalent interactions to promote BAR domain protein oligomerization on the membrane has not been investigated.

WASP is mutated in Wiskott-Aldrich syndrome (WAS) (*11*), which is associated with immunological deficiencies such as dermatitis, macrothrombocytopenia, and several other immune system disorders (*12*, *13*). Notably, the macrophages from patients with WAS exhibited defects in phagocytosis (*14*). Most of the mutations in WAS are within the WASP homology 1 (WH1) domain, thus affecting the interaction with WASP-interacting protein (WIP) and decreasing the stability of WASP (*15*, *16*). Other mutations are thought to affect the protein interactions at the Cdc42-Rac–interactive binding (CRIB) motif (*17*, *18*), the verprolin-homology, cofilin-homology, and acidic (VCA) domain, which activates the actin-related protein-2/3 (Arp2/3) complex (*19*), and PRR (*15*). The PRR contains a lot of proline-rich motifs (PRMs), and the PRR mutations of WASP in WAS apparently affected the cellular localization of WASP, although the binding partners that are affected by these PRR mutations have been unclear (*15*).

The PRR is involved in multivalent protein interaction. There are multiple SH3 domains in the adaptor proteins and the PRRs, leading to numerous protein interactions between them. These protein interactions were first reported between Nck and N-WASP, which resulted in the liquid-liquid phase–separated (LLPS) proteins in solution (*20*). The PRRs of WASP and N-WASP bind to the SH3 and WW domains (*21*–*23*). These SH3 domains are also within WISH/NCKIPSD/DIP/SPIN90, Nck, Grb2, Src, and Fyn, as well as the BAR domain–containing proteins (*10*), suggesting the possible regulation of the BAR domain–containing proteins by multivalent protein interaction. The presence of LLPS generally resulted in the disordered assembly, which caused the segregation in the cytoplasmic fluid. LLPS is also generated on the plasma membrane, downstream of receptors and adhesion molecules (*24*–*26*). However, it was unclear whether the LLPS or similar multivalent interactions contributed to the highly ordered assembly of the BAR domain superfamily proteins.

Phagocytosis is mediated by signaling cascades initiated by phagocytic receptors, including Fc-γ receptor II (FcγRII) (*27*, *28*). The cytoplasmic region of FcγRII has an immunoreceptor tyrosine activation motif (ITAM) that is phosphorylated by kinases, including Src family members, upon antibody binding in phagocytosis (*28*, *29*). The phosphorylated ITAM motif recruits the proteins with the Src homology 2 (SH2) domain. In addition, the guanine nucleotide exchange factors then activate Rho family guanosine triphosphatases (GTPases), including Cdc42, which are essential for the engulfment process of phagocytosis (*28*, *30*). In phagocytosis, Nck links WASP or N-WASP to the ITAM of the receptor (*31*). The activated Cdc42 binds to the WASP and the N-WASP (*32*, *33*). WISH is involved in the regulation of WASP and N-WASP, as well as the Arp2/3 complex (*34*, *35*). An analysis in the human reference protein interactome mapping project revealed that GAS7 also binds to WISH (*36*).

In this study, we developed fluorescence resonance energy transfer (FRET) monitoring to analyze the F-BAR–containing GAS7 oligomeric assembly on the membrane. We found that the highly ordered GAS7 oligomeric assembly is promoted by the multivalent interactions between WASP/N-WASP, Nck, and WISH, in the presence of the cytoplasmic region of the receptor FcγRII, Src kinase, and the activated Cdc42, on the lipid membrane. In contrast to LLPS, this GAS7 assembly was highly ordered and detected in macrophages under the activation of FcγRII in phagocytosis analysis.

## RESULTS

### FRET monitoring of oligomeric GAS7b assembly on membrane

**Fig. 1.**
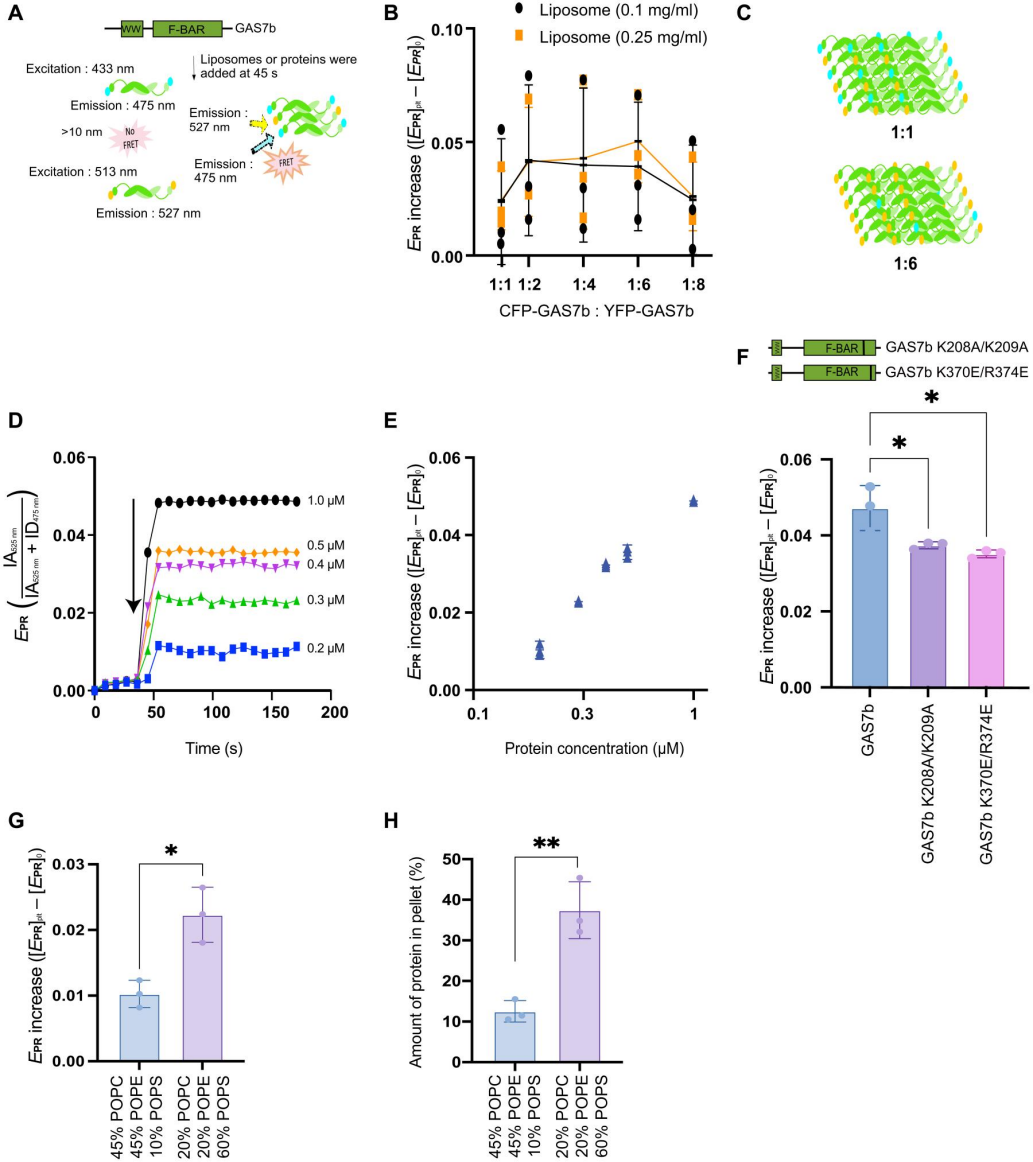
FRET of GAS7 with the membrane in vitro. (**A**) Illustration of the possible FRET of GAS7. (**B**) Relative increase in the FRET efficiency (*E*_PR_) of GAS7 (1 μM) with various ratios of CFP-GAS7b to YFP-GAS7b. Liposomes were made with 0.1 or 0.25 mg/ml of 20% POPC (1-palmitoyl-2-oleoyl-*sn*-glycero-3-phosphocholine), 20% POPE (1-palmitoyl-2-oleoyl-*sn*-glycero-3-phosphoethanolamine), and 60% POPS (1-palmitoyl-2-oleoyl-*sn*-glycero-3-phospho-l-serine) lipids. The means ± SD are shown (*n* = 3). (**C**) Illustration of the possible spatial distribution of GAS7b. (**D**) Time course of the *E*_PR_ upon the addition (arrow) of liposomes (0.25 mg/ml) composed of 45% POPC, 45% POPE, and 10% POPS to solutions of 0.2, 0.3, 0.4, 0.5, and 1 μM CFP-GAS7b and YFP-GAS7b at a ratio of 1:6. (**E**) Increase of the *E*_PR_ upon the addition of liposomes (0.25 mg/ml) to solutions containing 0.2, 0.3, 0.4, 0.5, and 1 μM CFP-GAS7b and YFP-GAS7b at a ratio of 1:6. The liposomes were composed of 45% POPC, 45% POPE, and 10% POPS. The means ± SD are shown (*n* = 3). (**F**) The increase of the *E*_PR_ of GAS7 (1 μM) and its K208A/K209A and K370E/R374E mutants in the presence of liposomes (0.25 mg/ml) composed of 45% POPC, 45% POPE, and 10% POPS. The means ± SD are shown (*n* = 3). (**G**) The increase of the *E*_PR_ of GAS7 (0.2 μM) in the presence of liposomes (0.25 mg/ml) composed of 45% POPC, 45% POPE, and 10% POPS or 20% POPC, 20% POPE, and 60% POPS. The means ± SD are shown (*n* = 3). (**H**) Liposome cosedimentation assay of GAS7 (0.2 μM), using the liposomes in (G). The amounts of proteins in the liposomal pellet are indicated. The means ± SD are shown (*n* = 3). The *P* values were obtained using one-way analysis of variance (ANOVA) with Dunnett’s post hoc analysis (F) and the two-tailed unpaired *t* test (G and H). Significance values are *P < 0.05 and **P < 0.01.

We monitored the oligomeric assembly of GAS7 by FRET. The fluorescence emission of the yellow fluorescent protein (YFP) upon the cyan fluorescent protein (CFP) excitation indicates the proximity localization of YFP to CFP within <10 nm (Fig. 1A) (*37*). GAS7b, with the WW domain and the F-BAR domain (Fig. 1A), is the splicing isoform that functions in phagocytosis by macrophages (*9*). We examined the FRET ratio between CFP-tagged GAS7b and YFP-tagged GAS7b (purification shown in fig. S1A) at various ratios. The liposomes containing 60% phosphatidylserine (PS) were thought to allow the binding of GAS7b by the negative charge of PS and thus were used as a membrane model that allows GAS7 binding without signaling factors. Tagged and nontagged GAS7b have similar binding characteristics to membranes containing various amounts of PS (fig. S1B), indicating that the protein tag did not affect the binding ability of GAS7b. The fluorescent emissions of FRET by CFP-GAS7b and YFP-GAS7b before and after the addition of liposomes showed the increase of YFP emission upon the addition of liposomes (fig. S1C). The FRET increased as the ratio of CFP-GAS7b to YFP-GAS7b increased, reaching a maximum at a protein ratio of 1:6 and decreasing at 1:8 (Fig. 1B). The maximum FRET at the 1: 6 ratio of CFP-GAS7b: YFP-GAS7b appeared to be compatible with the oligomeric sheet model of GAS7 assembly, where the probability of YFP in the vicinity of CFP was increased as the ratio increased (Fig. 1C) (*9*). These FRET observations strongly suggested the localization of GAS7b molecules in proximity to each other on the membrane.

Most of the plasma membrane contains PS, and its content is thought to be 10 to 20% (*38*, *39*). The addition of liposomes containing 10% PS to the CFP-GAS7b and YFP-GAS7b protein solution rapidly increased the FRET over the concentration range (Fig. 1D). The time resolution of our FRET measurement is ~9 s, and therefore, the lag time of GAS7b for its assembly appeared to be less than several seconds, if it existed. Upon the addition of liposomes containing 10% PS, the FRET efficiency increased as the protein concentration increased and did not appear to be saturated at 1 μM (Fig. 1, D and E). Furthermore, the K208A/K209A and K370E/R374E mutants of GAS7b have reduced binding to liposomes (*9*) and decreased the FRET efficiency significantly to the liposomes containing 10% PS (Fig. 1F). We modulated the interaction of GAS7b with the membrane by increasing the PS content in the liposomes. The FRET was higher with the liposomes containing 60% PS than that with 10% PS at 0.2 μM GAS7b (Fig. 1G). In vitro liposome cosedimentation assays have been widely used to demonstrate protein binding to liposomes. The binding at 0.2 μM GAS7b to the liposomes containing 10% PS was weaker than that to the liposomes containing 60% PS in the liposome cosedimentation assay (Fig. 1H). Therefore, the GAS7b binding to the membrane correlated with the FRET observations.

### Multivalent protein interactions of GAS7b binding proteins

**Fig. 2.**
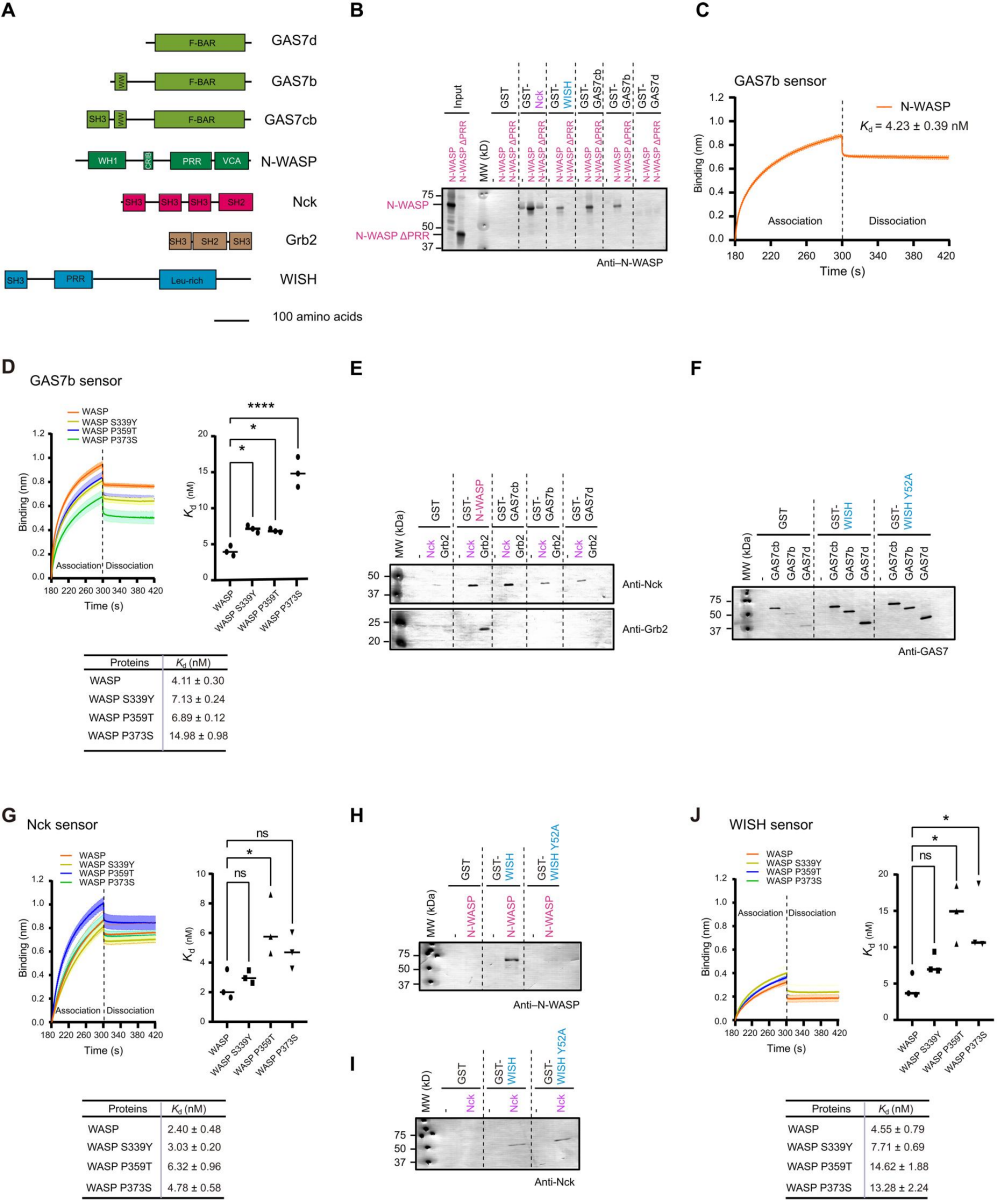
Protein-protein interactions of GAS7, N-WASP/WASP, Nck, and WISH. (**A**) Illustration of the domains of the proteins. Scale bar, 100 amino acid residues. (**B**) Binding of Nck, WISH, and GAS7 isoforms to N-WASP and its ΔPRR mutant. Nck, WISH, and GAS7 isoforms (1 μM) as GST fusion proteins were incubated with N-WASP and its ΔPRR mutant (0.5 μM). The bound proteins were analyzed by SDS–polyacrylamide gel electrophoresis, followed by Western blotting. GST was used as a negative control, and (−) indicates GST fusion protein alone. (**C** and **D**) Binding curve of immobilized GAS7b (0.1 μM) without GST to N-WASP (C) and WASP and its S339Y, P359T, and P373S mutants (D) by the biolayer interferometry analysis. The *K*_d_ values (*n* = 3) are shown on the right. The means ± SE are shown for the sensorgrams and *K*_d_ values (*n* = 3). (**E**) Binding of N-WASP and GAS7 isoforms to Nck and Grb2 (1 μM) as in (B). MW, molecular weight. (**F**) Binding of WISH and its Y52A mutant to splicing isoforms of GAS7 (0.5 μM) as in (B). (**G**) Binding curve of the immobilized Nck (0.1 μM) to WASP and its S339Y, P359T, and P373S mutants by the biolayer interferometry analysis, as in (D). (**H**) Binding of WISH and its Y52A mutant to N-WASP (0.5 μM) as in (B). (**I**) Binding of WISH and its Y52A mutant to Nck (0.5 μM) as in (B). (**J**) Binding curve of the immobilized WISH (0.1 μM) (H) to WASP and its S339Y, P359T, and P373S mutants by the biolayer interferometry analysis, as in (D). The *P* values were obtained using the one-way ANOVA with Dunnett’s post hoc analysis (D and G) and the Kruskal-Wallis test, followed by Dunn’s test (J). Significance values are *P < 0.05 and ****P < 0.0001. ns, not significant.

The phagocytotic cup is generated by the activation of signaling cascades, which include the Nck, N-WASP, WASP, WISH, GAS7b, Cdc42, and FcγRIIA proteins (Fig. 2A). The interaction of GAS7 with N-WASP and WASP has been described (*40*, *41*). However, the differences between the mouse GAS7d (human GAS7a, the F-BAR domain alone), mouse GAS7b (human GAS7b, the WW and F-BAR domains), and mouse GAS7cb (human GAS7c, the SH3, WW, and F-BAR domains) splicing isoforms in binding to N-WASP have not been determined. Furthermore, the binding was examined in different studies, and thus, the binding analyses under the same conditions, using the wild-type and mutant proteins, were required to understand the overlapping or nonoverlapping binding modes between these proteins for possible multivalent assembly.

We prepared the glutathione *S*-transferase (GST) fusion proteins and the proteins without a tag for pull-down assays (fig. S1A). N-WASP was found in the pellet in the pull-down assays using GAS7cb and GAS7b, but not that using GAS7d, indicating the binding of GAS7cb and GAS7b to N-WASP (Fig. 2B and fig. S2A). The binding of N-WASP to GAS7cb appeared to be higher than that to GAS7b. Because the SH3 and WW domains bind to PRR, the binding of N-WASP to GAS7 was suggested to occur between the SH3 and/or WW domains of GAS7 and the PRR of N-WASP (Fig. 2B and fig. S2B). Accordingly, the deletion of the PRR diminished the binding in the pull-down assay (Fig. 2B). To examine the binding of these proteins more quantitatively, without the potential inaccuracy from the GST fusion proteins, we used the biolayer interferometry analysis to monitor the binding of monomeric Avi-tagged protein on the glass surface. The dissociation constant (*K*_d_) between GAS7b and N-WASP by the kinetic measurement was 4.23 ± 0.39 nM (Fig. 2C).

We next examined the binding of GAS7b, the isoform expressed in macrophages, to WASP by the biolayer interferometry analysis as mentioned above. The *K*_d_ value between GAS7b and WASP was 4.11 ± 0.30 nM (Fig. 2D and fig. S2C). The WAS disease mutations in the PRR altered the affinity between GAS7b and WASP. The biolayer interferometry analyses showed that the *K*_d_ values between GAS7b and the S339Y, P359T, and P373S mutants of WASP were 7.13 ± 0.24 nM, 6.89 ± 0.12 nM, and 14.98 ± 0.98 nM, respectively (Fig. 2D and fig. S2C), which are quite different from that between GAS7b and WASP. These results indicated that the PRRs of WASP and N-WASP are the binding sites for GAS7b.

Grb2 and Nck are adaptor proteins that bridge the phosphorylated tyrosine and PRMs (*42*–*44*). Grb2 and Nck contain two and three SH3 domains, respectively. WISH is also an adaptor protein for N-WASP and WASP. In the pull-down assay, Grb2 bound to N-WASP but not to GAS7 isoforms (Fig. 2E and fig. S2D). Nck interacted with all isoforms of GAS7. These assays revealed that WISH could bind to all GAS7 isoforms (Fig. 2F and fig. S2E). The binding affinities of Nck and WISH to GAS7b and GAS7d were similar (Fig. 2, E and F), suggesting a possible binding site in the F-BAR domain (Fig. 2A).

The binding abilities of the GAS7 F-BAR domain mutants of K208A/K209A and the hydrophilic loop (amino acids 206 to 219, FFL2) deletion were examined for N-WASP, WISH, and Nck. The pull-down assay revealed that the hydrophilic loop deletion and K208A/K209A mutation reduced the binding of GAS7b to Nck and WISH but not to N-WASP (fig. S2F).

We then examined Nck binding to WASP and N-WASP. The PRR deletion diminished the binding, confirming that it mediates Nck binding (*20*). Biolayer interferometry analyses showed that the *K*_d_ values between Nck and WASP and its S339Y, P359T, and P373S mutants were 2.40 ± 0.48 nM, 3.03 ± 0.20 nM, 6.32 ± 0.96 nM, and 4.78 ± 0.58 nM, respectively (Fig. 2G and fig. S2C). The binding mode of WASP to Nck was significantly different from that of the P359T mutant.

The interaction of WISH with N-WASP was previously reported (*22*, *45*), but that with WASP had not. Therefore, we examined the binding of WISH to WASP and N-WASP. The pull-down assay revealed that the N-WASP binding to the WISH Y52A mutant in the SH3 domain was weaker than that to WISH (Fig. 2H and fig. S2G). Accordingly, the PRR of N-WASP mediated the WISH binding, because its deletion reduced the WISH binding to N-WASP in the pull-down assay (Fig. 2B). These results suggested that the WISH Y52A mutant did not interact with N-WASP. The Y52A WISH mutant had similar affinities to GAS7 isoforms and Nck (Fig. 2, H and I, and fig. S2, E and H), suggesting that its PRR could bind to GAS7 and Nck.

We also examined the binding affinities of WASP and its S339Y, P359T, and P373S mutants to WISH. Biolayer interferometry analyses revealed that the *K*_d_ values between WISH and WASP and its S339Y, P359T, and P373S mutants were 4.55 ± 0.79 nM, 7.71 ± 0.69 nM, 14.62 ± 1.88 nM, and 13.28 ± 2.24 nM, respectively (Fig. 2J). There is a significant difference in the binding of WASP to WISH from those of the WASP P359T and P373S mutants, suggesting that the SH3 domain of WISH is bound to the PRR.

Together, these mutant binding data indicated that WISH and its Y52A mutant have different binding modes to WASP/N-WASP, but not to Nck or GAS7b, whereas WASP and its mutants have different binding modes to GAS7b and WISH but not to Nck. Therefore, they bind at different sites from each other, indicating the formation of multivalent complexes.

### Increased GAS7b assembly by multivalent binding

**Fig. 3.**
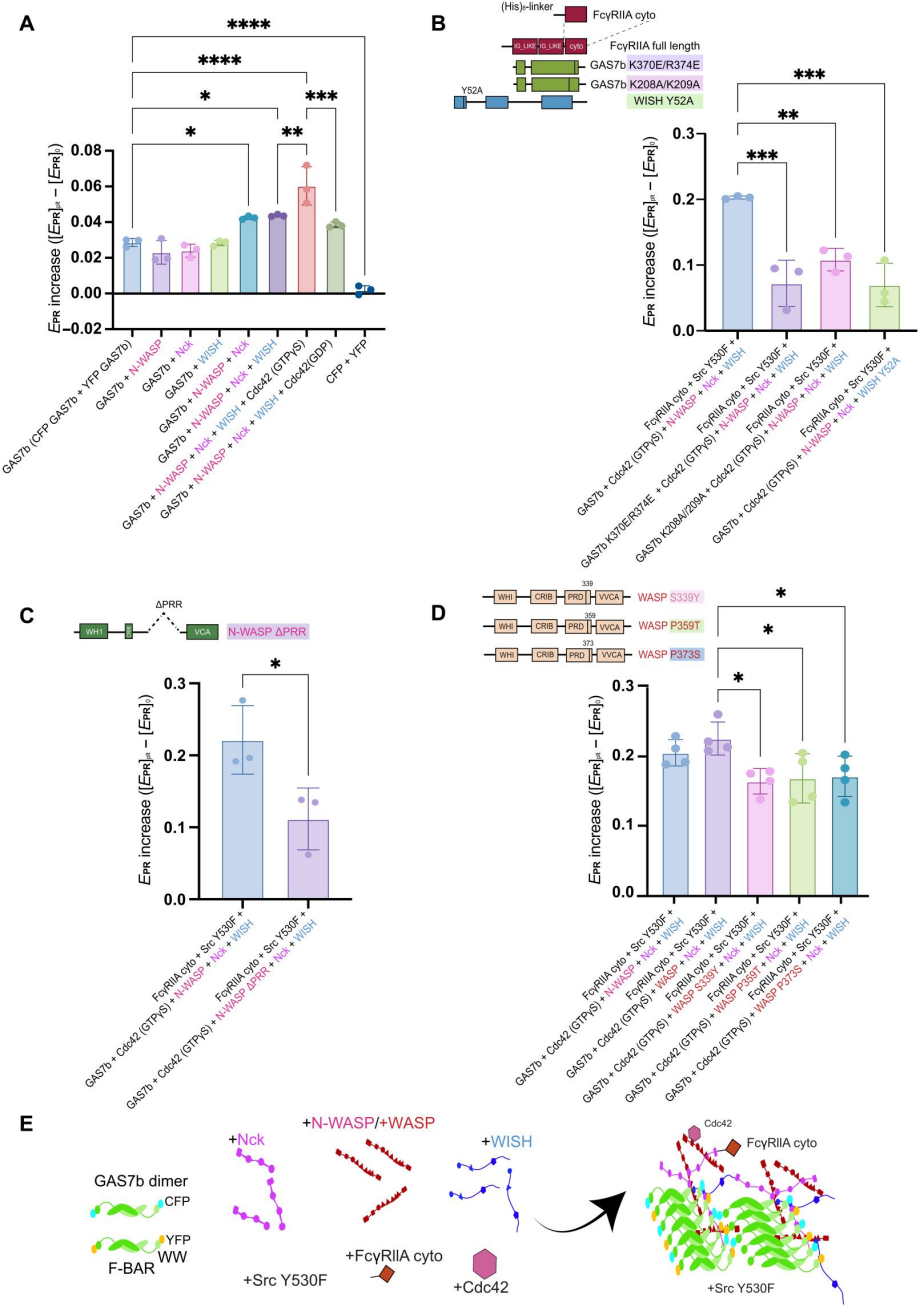
Increase of the FRET of GAS7 with the membrane and the multivalent proteins. (**A**) Relative FRET efficiency (*E*_PR_) upon the addition of liposomes (0.25 mg/ml of 45% POPC, 45% POPE, and 10% POPS) to solutions containing CFP and YFP (1 μM), CFP-GAS7b, and YFP-GAS7b (1 μM), N-WASP (0.5 μM), Nck (0.5 μM), WISH (0.5 μM), and/or FLAG-tagged Cdc42(GTPγS or GDP loaded) (0.1 μM). (*n* = 3). (**B**) Increase of the *E*_PR_ upon the addition of the protein solution to liposomes (0.25 mg/ml of 45% POPC, 45% POPE, 10% POPS, and 10% DGS-NTA) with 0.05 μM His-tagged FcγRIIA cytoplasmic region (cyto) and 0.025 μM Src Y530F. The protein solution contained CFP-GAS7b and YFP-GAS7b or its K208A/K209A or K370E/R374E mutants (1 μM), WISH or its Y52A mutant (0.5 μM), N-WASP (0.5 μM), Nck (0.5 μM), and Cdc42(GTPγS) (0.1 μM). (*n* = 3). (**C**) Increase of the *E*_PR_ upon the addition of the protein solution to liposomes (0.25 mg/ml of 45% POPC, 45% POPE, 10% POPS, 10% DGS-NTA, and 5% PIP_2_) with 0.05 μM His-tagged FcγRIIA cytoplasmic region (cyto) and 0.025 μM Src Y530F. The protein solution contained CFP-GAS7b and YFP-GAS7b (1 μM), WISH (0.5 μM), N-WASP or its ΔPRR mutant (0.5 μM), Nck (0.5 μM), and Cdc42(GTPγS) (0.1 μM) (*n* = 3). (**D**) Increase of the *E*_PR_ as in (C). The protein solution contained CFP-GAS7b and YFP-GAS7b (1 μM), WISH (0.5 μM), N-WASP (0.5 μM), or WASP and its S339Y, P359T, and P373S mutants (0.1 μM), Nck (0.5 μM), and Cdc42(GTPγS) (0.1 μM) (*n* = 4). (**E**) Illustration of possible multivalent interactions. The *P* values were obtained by the two-tailed unpaired *t* test (C) and one-way ANOVA with Tukey’s (A) and Dunnett’s (B and D) post hoc analyses. The means ± SD are shown. Significance values are *P < 0.05, **P < 0.01, ***P < 0.001, and ****P < 0.0001.

To understand how the multivalent protein interactions affect the GAS7b assembly, we measured the FRET efficiency of GAS7 upon the addition of liposomes in the presence of the proteins, as above (Fig. 2A). We used liposomes containing 10% PS. The addition of these liposomes increased the FRET efficiency by GAS7b alone (Fig. 3A). The FRET was not significantly changed in the presence of either N-WASP, Nck, or WISH as compared with GAS7b alone (Fig. 3A). The presence of N-WASP, Nck, and WISH together significantly changed the FRET efficiency of GAS7b (Fig. 3A).

WASP and N-WASP interact with an active form of Cdc42. Therefore, we prepared Cdc42 with lipid modifications from mammalian cells, and the nonhydrolyzable analog of GTP, GTPγS, was loaded onto Cdc42. The presence of GTPγS-loaded Cdc42 [Cdc42(GTPγS)], together with N-WASP, Nck, and WISH, enhanced the FRET efficiency of GAS7b, while the presence of guanosine diphosphate (GDP)–loaded Cdc42 [Cdc42(GDP)] or *Escherichia coli*–derived Cdc42 without lipid modifications did not (Fig. 3A and fig. S3). Therefore, an active form of Cdc42 with lipid modifications promoted the GAS7 assembly on the liposomes.

The Cdc42 activation in phagocytosis relates to the phagocytotic receptor FcγRIIA, which is phosphorylated by Src and then supposed to recruit Nck, followed by N-WASP (*31*). The cytoplasmic region of FcγRIIA (FcγRIIA cyto) was purified with a His-tag and then attached to liposomes containing nickel-conjugated lipids {1,2-dioleoyl-*sn*-glycero-3-[(*N*-(5-amino-1-carboxypentyl)iminodiacetic acid)succinyl] (DGS-NTA)}. FcγRIIA cyto was phosphorylated by the active form of Src, the Src Y530F mutant, before the addition of GAS7b and/or Cdc42, N-WASP, Nck, and WISH. The FRET efficiency in the presence of Cdc42, N-WASP, Nck, and WISH was examined and found to be enhanced in the presence of liposomes with the phosphorylated FcγRIIA cyto (Fig. 3B). To examine the specificity of this FRET increase, the mutants that disrupt these interactions between proteins and membranes were tested for the FRET of GAS7. The FRET efficiencies of the K208A/K209A and K370E/R374E mutants of GAS7b without membrane binding were smaller than that of GAS7b (Fig. 3B). The FRET efficiency was also decreased in the presence of the WISH Y52A mutant in the SH3 domain (Fig. 3B).

We then examined the protein assembly by FRET in the presence of PIP_2_ (phosphatidylinositol 4,5-bisphosphate), which is essential for phagocytosis, in liposomes composed of 45% POPC (1-palmitoyl-2-oleoyl-*sn*-glycero-3-phosphocholine), 45% POPE (1-palmitoyl-2-oleoyl-*sn*-glycero-3-phosphoethanolamine), 10% POPS (1-palmitoyl-2-oleoyl-*sn*-glycero-3-phospho-l-serine), 10% DGS-NTA, and 5% PIP_2_ (Fig. 3, C and D). The liposomes with PIP_2_ also increased the FRET efficiency of GAS7 (Fig. 3, C and D). The importance of multivalent protein interactions was examined by using N-WASP with the PRR deletion and the WASP disease mutants at the PRR. The ΔPRR mutant of N-WASP exhibited reduced FRET efficiency as compared to that of N-WASP (Fig. 3C). The FRET in the presence of the S339Y, P359T, and P373S WASP mutants was significantly decreased as compared to that in the presence of WASP (Fig. 3D). These results indicated that GAS7b and the interactive proteins could be recruited onto the membrane upon the activation of receptors through the multivalent protein interactions, as illustrated in Fig. 3E.

### GAS7b assembly on giant liposomes by multivalent protein interactions

The above FRET analyses indicated the GAS7b assembly on the membrane; however, microscopic observations are essential to verify the assembly. Therefore, we examined the protein assembly on giant unilamellar vesicles (GUVs) containing 10% PS, the same composition as that in the FRET experiment, and observed them by microscopy. GAS7b was localized homogeneously on these GUVs, and the biased localization was only observed on small percentages of GUVs (fig. S4, A and B). The presence of Cdc42, N-WASP, Nck, and WISH slightly but significantly promoted the biased localization of GAS7b (fig. S4, A and B).

**Fig. 4.**
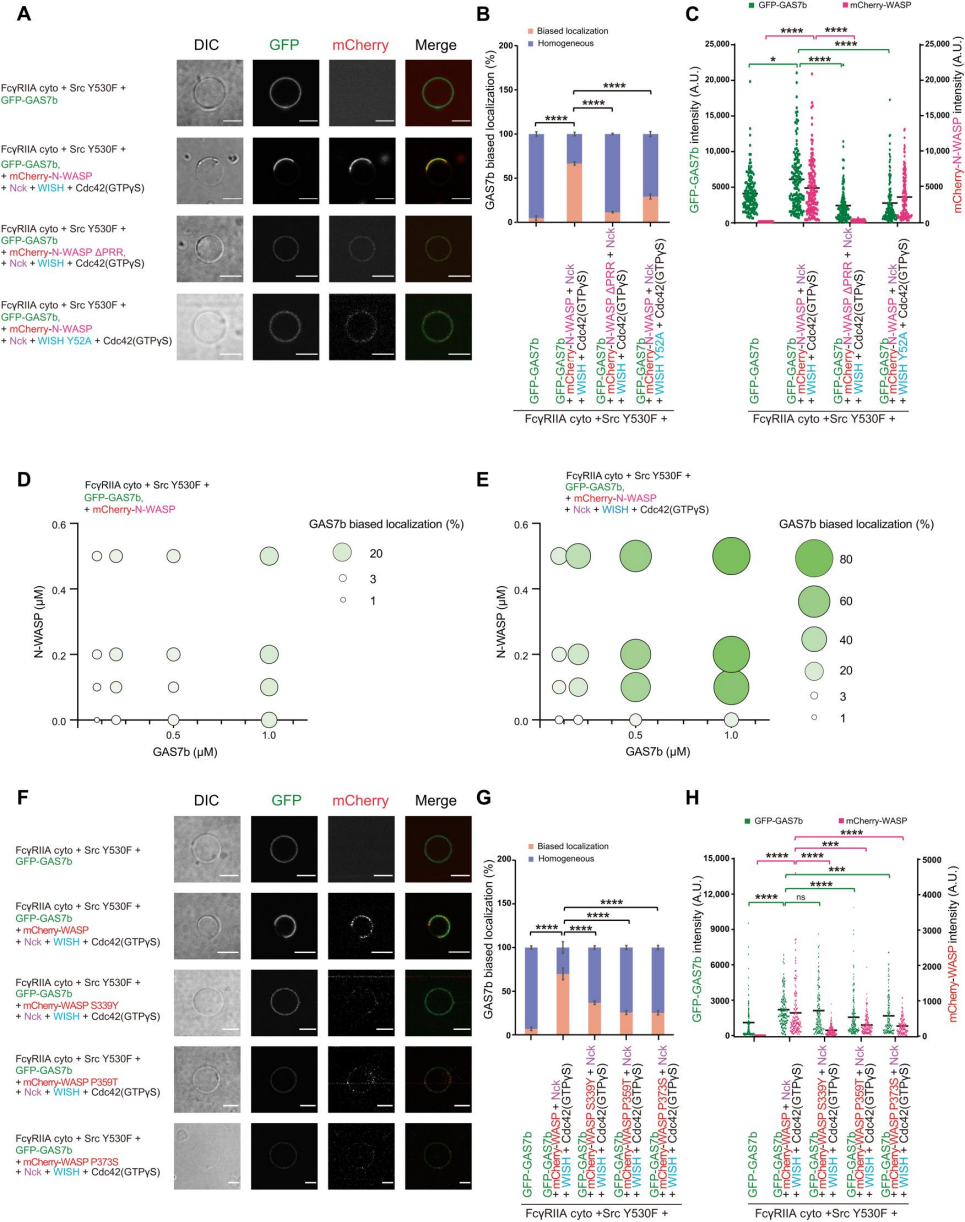
Protein localization on the GUVs. (**A**) Representative images of GUVs (0.1 mM lipids containing 45% POPC, 45% POPE, 10% POPS, 10% DGS-NTA, 5% PIP_2_, and 0.5% biotin-PE) incubated with 0.05 μM His-FcγRIIA cyto and 0.025 μM Src Y530F, visualized by differential interference contrast (DIC) and their associated proteins by the GFP and mCherry fluorescence. The proteins were 1 μM GFP-GAS7b, 0.5 μM mCherry-N-WASP or its ΔPRR mutant, 0.5 μM Nck, 0.5 μM WISH or its Y52A mutant, and 0.1 μM Cdc42(GTPγS). (**B**) Frequency of GUVs with the biased localization of GFP-GAS7b in (A). (**C**) Intensities of GFP and mCherry fluorescence on a GUV in (A). The sums of the intensities are plotted with the means in arbitrary units (A.U.). (**D**) Diagram of the frequency of GUVs with the biased protein localization of GFP-GAS7b, using various concentrations of GFP-GAS7b and mCherry–N-WASP in the presence of 0.05 μM His-FcγRIIA cyto and 0.025 μM Src Y530F. Each circle in the diagram represents the frequency of the biased localization. (**E**) Diagram showing the frequency of GUVs as in (D) upon the addition of 0.5 μM Nck, 0.5 μM WISH, and 0.1 μM Cdc42(GTPγS). (**F**) Representative images of GUVs as in (A). The proteins were 1 μM GFP-GAS7b and 0.1 μM mCherry-WASP or its S339Y, P359T, and P373S mutants, 0.5 μM Nck, 0.5 μM WISH, 0.1 μM Cdc42(GTPγS), 0.05 μM His-FcγRIIA cyto, and 0.025 μM Src Y530F. (**G**) Frequency of GUVs with the biased localization of GFP-GAS7b in (F). (**H**) Intensities of fluorescence on a GUV in (F) as in (C). The *P* values were obtained by one-way ANOVA with Dunnett’s post hoc analysis (B and G) the Kruskal-Wallis test, followed by Dunn’s test (C and H). Significance values are *P < 0.05, ***P < 0.001, and ****P < 0.0001. Scale bars, 5 μm.

Then, we prepared GUVs decorated with the cytoplasmic region of His-tagged FcγRIIA, which was anchored to the liposomes via DGS-NTA and phosphorylated by the Y530F mutant of Src. The localization of GAS7b alone to these liposomes was similar to that without FcγRIIA (Fig. 4A and fig. S4A). GAS7b at various concentrations was localized homogeneously on these GUVs (Fig. 4, A to C), and the addition of N-WASP alone slightly changed the degree of the biased localization (Fig. 4D). The addition of WISH, Nck, Cdc42, and N-WASP resulted in the appearance of biased protein localization in GUVs containing GAS7b and N-WASP (Fig. 4, A to C). The biased localization was dependent on the concentrations of GAS7b and N-WASP in the presence of WISH, Nck, and Cdc42, suggesting a condensed protein assembly by multivalent interactions (Fig. 4E). Furthermore, the PRR deletion mutant of N-WASP and the WISH Y52A mutant in the SH3 were not able to generate the biased localization of GAS7b on the membrane (Fig. 4, A to C). In contrast, the PRR deletion mutant of N-WASP and the WISH Y52A mutant, instead of their wild-type proteins, diminished the protein recruitment to the GUVs (Fig. 4, A to C). Quantification of the fluorescence intensities of GFP-GAS7b and mCherry-N-WASP indicated the enhanced recruitment of these proteins onto GUVs (Fig. 4C). The presence of WASP also supported the biased localization of GAS7b, in a similar manner to N-WASP (Fig. 4, F to H). Accordingly, the PRR mutants of WASP decreased the concentrated localization of GAS7b, altering it to a homogeneous status on the membrane (Fig. 4, F to H). Therefore, the biased localization of the proteins is considered to be a condensed protein assembly as a result of multivalent protein interactions through the PRR and the SH3 domains.

### Highly ordered GAS7 assembly on the membrane

**Fig. 5.**
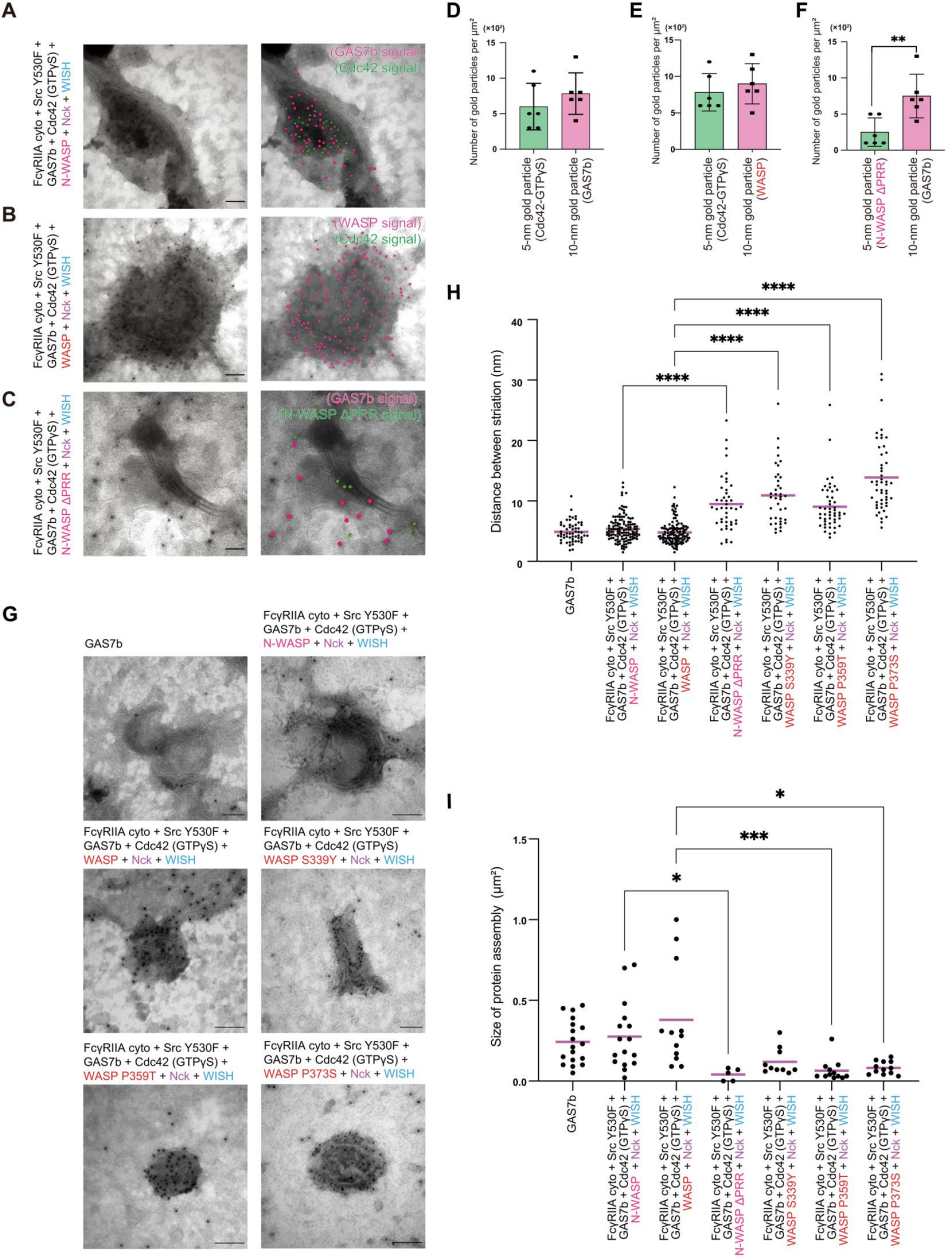
Striations of the protein assembly with GAS7b on the membrane. (**A**) Representative transmission electron microscopic images of the immuno-gold labeling of proteins on a monolayered membrane incubated with 0.1 μM GAS7b, 0.1 μM N-WASP, 0.1 μM Nck, 0.1 μM WISH, 0.1 μM Cdc42(GTPγS), 0.1 μM FcγRIIA cyto, and 0.1 μM Src Y530F. The 5- and 10-nm gold particles are for Cdc42 and GAS7b, respectively. The raw image is shown on the left, and the 5-nm (green) and 10-nm (magenta) gold particles are marked in the right image. The monolayer membrane was composed of 45% POPC, 45% POPE, 10% POPS, 10% DGS-NTA, and 5% PIP_2_. (**B**) Representative transmission electron microscopic image of the immuno-gold labeling of proteins as in (A), with WASP instead of N-WASP. The 5- and 10-nm gold particles are for Cdc42 and WASP, respectively. (**C**) Representative transmission electron microscopic image of the immuno-gold labeling of proteins as in (A), with the N-WASP ΔPRR mutant instead of N-WASP. The 5- and 10-nm gold particles are for N-WASP and GAS7, respectively. (**D** to **F**) Densities of the 5- and 10-nm gold particles in the protein assemblies in (A) to (C), respectively. The density per micrometer squared (*n* = 6) is shown. (**G**) Representative transmission electron microscopic image of the immuno-gold labeling of proteins as in (A), but WASP and its mutants were included instead of N-WASP. The 10-nm gold particles are linked to the GAS7 antibody and identified the area covered with GAS7. (**H**) Frequencies of the distances between striations. The lines represent means. (**I**) Frequencies of protein assembly sizes quantified from the protein-decorated areas. The lines represent means. The *P* values were obtained using the Kruskal-Wallis test, followed by Dunn’s test. Significant values are *P < 0.05, **P < 0.01, ***P < 0.001, and ****P < 0.0001. Scale bars, 100 nm.

The multivalent protein interactions between the PRR and the multiple SH3 domain proteins are thought to generate disordered protein assemblies. However, we previously reported that the GAS7 F-BAR domain assembled on the membrane in ordered striations (*9*). The FRET of GAS7 here also suggested that the defined distance between the GAS7 molecules assembled on the membrane. Therefore, the striation of GAS7b was examined on a flat monolayered membrane by transmission electron microscopy. The GAS7b striations were detected in the presence of Cdc42, N-WASP, Nck, and WISH (fig. S5A). These membrane striations were also observed with the cytoplasmic region of FcγRIIA and the Src Y530F mutant (Fig. 5, A to C and fig. S5B). To determine whether the striations contained Cdc42, N-WASP, or WASP, together with GAS7b, we used the antibodies for these proteins with visualization by 5- or 10-nm gold particles in the secondary antibodies (Fig. 5, D to F). Cdc42 was also present in the protein assemblies on the monolayered membrane (Fig. 5, A, B, D, and E). WASP also supported similar striations as N-WASP (Fig. 5, B and E). The ΔPRR mutant of N-WASP did not assemble with GAS7b and appeared to inhibit the GAS7b assembly (Fig. 5F).

We then examined the effects of PRR mutations on these assemblies on the monolayered membrane (Fig. 5G). The distances between striations in the presence of the ΔPRR mutant of N-WASP and the WAS PRR mutants were wider than those in the presence of WASP and N-WASP (Fig. 5H). The size of the assemblies in the presence of Cdc42, N-WASP, Nck, and WISH together with FcγRIIA and the Src Y530F were sometimes enlarged (Fig. 5I). In contrast, the P359T and P373S mutants of WASP and the ΔPRR mutant of N-WASP exhibited smaller GAS7 assemblies than those in the presence of WASP or N-WASP (Fig. 5I), indicating that the PRRs of WASP and N-WASP are involved in determining the size and order of the protein assembly by the multivalent interactions. Therefore, the GAS7b assembly contained and was dependent on N-WASP or WASP and Cdc42.

### Cellular GAS7b assembly in frustrated phagocytosis

**Fig. 6.**
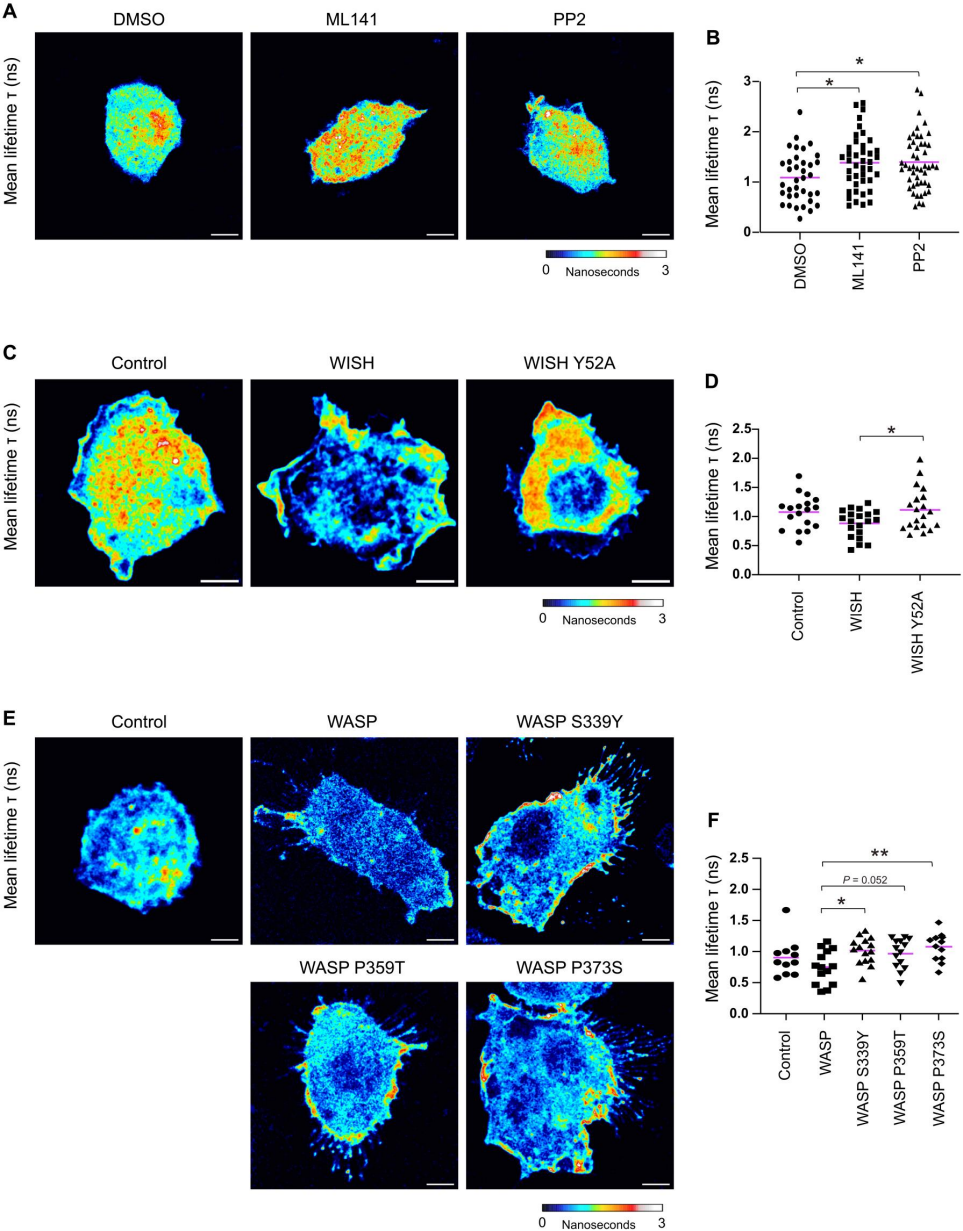
FRET of GAS7 in macrophage cells under frustrated phagocytosis. (**A**) Representative images of RAW 264.7 macrophage cells expressing YFP-GAS7b-IRES-CFP-GAS7b under frustrated phagocytosis. The cells expressed mCherry before the treatments with 0.1% DMSO (control), 10 μM ML141 (Cdc42 inhibitor), and 10 μM PP2 (Src inhibitor) for 20 min at 37°C. The pseudo colors represent the fluorescent lifetimes of CFP. (**B**) Plots of the mean lifetime τ (nanoseconds) of CFP of (A). The lines indicate the means. (**C**) Representative image of RAW 264.7 macrophage cells under frustrated phagocytosis, as in (A). The cells expressed mCherry, mCherry-WISH, or mCherry-WISH Y52A. (**D**) Plots of the mean lifetimes τ (nanoseconds) of CFP of (C). (**E**) Representative image of RAW 264.7 macrophage cells under frustrated phagocytosis, as in (A). The cells expressed mCherry, mCherry-WASP, mCherry-WASP S339Y, mCherry-WASP P359T, or mCherry-WASP P373S. (**F**) Plots of the mean lifetimes τ (nanoseconds) of CFP of (E). The *P* values were obtained by one-way ANOVA with Dunnett’s post hoc analysis. Significant values are *P < 0.05 and **P < 0.01. Scale bars, 5 μm.

The highly ordered, striated GAS7b assembly was strongly indicated to be in the phagocytotic cup of macrophage RAW246.7 cells (*9*). We performed fluorescence lifetime imaging (FLIM) to monitor the FRET in cells undergoing frustrated phagocytosis, where the cells spread their cell membrane onto immunoglobulin G (IgG)–coated glass as trying to engulf it (the frustrated phagocytosis) (*46*). We first transformed GAS7 knockout RAW264.7 cells with a bi-cistronic vector expressing both YFP-GAS7b and CFP-GAS7b in a constant ratio. Next, we measured the fluorescent lifetime of CFP, because it changes dependent on the degree of FRET. We then tested the contributions of Cdc42 and Src kinases to the FRET by treating the cells with inhibitors of Cdc42 (ML141) and Src (PP2) and compared the values to those of the cells treated with their carrier solvent, dimethyl sulfoxide (DMSO), as a negative control (Fig. 6A). The fluorescence lifetimes of CFP of the cells treated with ML141 and those treated with PP2 were significantly longer as compared to those of DMSO-treated cells, suggesting that the inactivation of the Cdc42 and Src kinases significantly reduced the GAS7 assembly in the cells (Fig. 6, A and B).

In macrophages, WASP is dominantly expressed over N-WASP. We next examined whether the ectopic expression of mutants of WASP and WISH that affected the multivalent assembly also affected the fluorescence lifetime of CFP-GAS7b in the cells. The cells expressing the Y52A mutant of WISH exhibited a longer fluorescence lifetime than that of the cells expressing WISH (Fig. 6, C and D). The fluorescent lifetimes of the S339Y, P359T, and P373S mutants of WASP-expressing cells were significantly longer than that of the WASP-expressing cells (Fig. 6, E and F). These results revealed that the GAS7 assembly was significantly weakened by the expression of these mutants, indicating that the multivalent interactions in vitro functioned in the frustrated phagocytosis.

## DISCUSSION

The BAR domain superfamily proteins oligomerize into highly ordered assembly on the membrane. However, the regulation of their oligomerization in the ordered configuration of GAS7 remained enigmatic. In this study, we found that the activation of Cdc42 and the Fcγ receptor can regulate the assembly of the F-BAR protein GAS7 into the ordered striations, depending on multivalent interactions that involve the WASP family proteins and the adaptor proteins.

### Multivalent interactions for the assembly of BAR domain–containing proteins

In the biological system, the multivalent interaction through LLPS that forms the dense phase, known as biomolecular condensates or scaffolds, is one of the major mechanisms of the assemblies of proteins. The formation of multivalent assemblies by the WASP family proteins and the scaffold proteins downstream of receptors has been reported (*20*, *47*, *48*); however, the involvement of WASP family proteins for the assembly of the BAR domain–containing proteins has not been reported before.

Most of the multivalent protein assembly, at least in vitro, requires the slower diffusion of molecules for crowding, which is achieved by the addition of polyethylene glycol (PEG) in most of the in vitro studies (*20*, *49*). Among BAR domain–containing proteins, the condensation of endophilin (N-BAR protein) at the membrane by the proline-rich motifs of lamellipodin (LPD-PRMs) (*50*) and of IRSp53 (I-BAR protein) by the PDZ domain of postsynaptic density–95 (PSD-95) protein, which specifically binds to the IRSp53 (*51*) were reported. In these studies, the BAR domains did not appear to be ordered. However, the BAR domains are considered to form lattice-like, highly ordered assemblies for the membrane shaping (*52*).

In this study, the highly ordered GAS7 assembly by multivalent interactions on the membrane was achieved with lipid membranes without the use of PEG. The interactions of GAS7b on the membrane occur without multivalent interactions. However, the multivalent interaction altered GAS7 assembly into the locally concentrated, biased localization with N-WASP/WASP, Nck, WISH, and Cdc42. Thus, the two-dimensional environment of the membrane may provide a diffusion limitation, enhancing the multivalent protein assembly.

### Cellular signaling for the protein condensates at the membrane

Activation of Cdc42 was reported to serve as a crucial step in various cellular phenomena. However, the regulation of the multivalent protein assembly for phase-separated condensates has been unclear. Among the Cdc42 binding proteins, WASP, N-WASP, PAR protein complex, and GRK- interactor ARF GAP (GIT) / PAK-interacting exchange factor (PIX) have been studied in terms of protein assembly by multivalency (*53*, *54*). However, the role of Cdc42 activation in the assembly of the BAR domain superfamily proteins, including GAS7, has remained unclear. In this study, the activated Cdc42 was found to enhance the F-BAR protein GAS7 assembly in the presence of scaffold proteins. WASP and N-WASP are the only proteins binding Cdc42 in our in vitro analysis, and thus, the membrane-anchored Cdc42 is thought to recruit these proteins to the membrane.

Among the receptors, the linker of activated T cells and nephrin receptors reportedly promote the formation of multivalent assemblies (*20*, *47*). The “T cell” receptor signaling cluster and transcriptional coactivators phase separate into protein condensates, which is mediated by the multivalent binding of SH3, SH2 (*47*), and WW domains (*55*, *56*). In this study, the phosphorylated FcγRIIA was tethered on the membrane, promoting the multivalent assembly of GAS7 through the recruitment of scaffold proteins. Therefore, this study has successfully reconstituted the signaling pathway for the assembly of GAS7.

### WASP family as a regulator of the BAR domain superfamily

N-WASP and WASP are proteins that were first studied for their multivalent assembly capabilities (*20*, *57*). There are a lot of mutations in the WAS. However, the mechanisms of WAS mutation at the PRR for the defects in WASP function have been unclear. In our study, the WAS mutations at the PRR altered the binding to GAS7b, Nck, and WISH, thus changing the shape of the GAS7 assembly and the decrease of biased localization on the membrane and in the cells. Therefore, the altered function of WASP by WAS mutation at the PRR is found to be the alteration in multivalent protein assembly here. The WAS mutation at the PRR is only a single-point mutation. Therefore, the multivalent binding of WASP was suggested to be altered by a loss of single binding sites. The WAS mutations might alter the protein interaction network into a nonfunctional state–like protein aggregation (*58*).

The assembly mechanism of GAS7 is likely to suggest the assembly mechanisms of other BAR domain superfamily proteins. The key players of the GAS7 assembly, i.e., WASP family proteins, the adaptor proteins, and the small GTPase, are also the binding partners for the BAR domain superfamily proteins (*10*). Therefore, other BAR domain assemblies may also be strongly suggested to be regulated in a similar manner.

### The assembly of the F-BAR domain protein

The BAR domain superfamily proteins form oligomers on the membrane. The oligomer surface of the BAR domains is defined, and thus, the assembly is highly ordered, although they are unique to each other (*4*, *9*, *59*). Such an ordered assembly is found in the polymerization of the cytoskeletal proteins. Nucleation is an initial stage in the assembly of polymeric structures, including those of actin, microtubules, intermediate filaments, and septins. The polymerization of actin and tubulin requires nucleotide binding to the monomers as well as the nucleation core of polymerization. The nucleation core of actin polymerization is the trimer of actin, the exposure of the free barbed end or the activated Arp2/3 complex. Previous studies show that actin nucleation by the Arp2/3 complex was initiated by a nucleating factor like the WASP protein family (*10*, *19*, *60*, *61*), resulting in the shortening of a lag time for the onset of actin polymerization. In contrast, septin and intermediated filament proteins do not require nucleotide binding for oligomerization. The intermediates filament vimentin forms tetramer and then oligomerizes into filaments (*62*, *63*). Septin can polymerize without any lag phase (*64*, *65*). Septins also bind to the membrane as septin oligomers and deform it (*66*, *67*). The BAR domain superfamily proteins, including GAS7, are not thought to bind to nucleotides because of the lack of such structural signatures. The kinetics of the GAS7 assembly, i.e., the F-BAR domain assembly, did not appear to have a lag time in the time resolution of 9 s here and appeared to be triggered by the addition of liposomes in our concentration range. According to the FRET efficiency, the assembly reached the maximum in seconds after the addition of liposomes, and therefore, it would be speculated that the onset of the GAS7 assembly would be rapid enough for the cells to change their shapes in response to stimuli.

In this study, the GAS7 was shown to assemble into a large and highly ordered protein scaffold together with the adaptor proteins. Therefore, such BAR domain assembly on the membrane is the scaffold for their binding proteins to locally accumulate around the receptors, thereby providing a possible platform for the signaling proteins beneath the membrane. Then, the signaling proteins are thought to enlarge the assembly of the BAR domain proteins in highly ordered manners for shaping the cellular membranes.

## MATERIALS AND METHODS

### Plasmid construction

The mouse GAS7 (GenBank accession, XM_006532202.1) constructs were described previously (*9*). Various point mutations, deletions, and substitutions of GAS7b were generated by polymerase chain reaction (PCR). The enhanced green fluorescent protein (EGFP)–GAS7b fusion was described previously (*9*) and subcloned into the pGEX6P-1 vector (GE Healthcare). The CFP and YFP were replaced with EGFP. N-WASP (GenBank accession, NM_174219.2) was subcloned into the pGEX6P-1 vector. N-WASP ΔPRR was deleted at amino acid residues 272 to 394. WISH (*22*), Nck (*68*), Grb2 (GenBank accession, NM_030846.2), His-FcγRIIA cytoplasmic region (cyto), and Cdc42 (GenBank accession, NM_044472.3) were also subcloned into the pGEX6P-1 vector. The WISH Y52A mutant was generated by PCR. WASP (GenBank accession, NP_000368.1) and its S339Y, P359T, and P373S mutants were subcloned into the pCMV-T2B vector with a FLAG tag (Stratagene), and Src Y530F 3xFLAG was cloned into the pΔEGFP-N1 vector in which the EGFP was removed from the pEGFP-N1 vector. WASP were also subcloned into the pmCherry-C1 vector (Clontech). N-WASP was subcloned into pGEX6P-1 (GE Healthcare) with inserted mCherry. pEGFP-C1-GST-Cdc42 was generated by subcloning the Cdc42 in pGEX6P-1. The genes encoding the C-terminal Avitag-fused GAS7b, Nck, and WISH were cloned into pGEX6P1 expression vectors. All plasmids were cloned using Gibson assembly, transformed into *E. coli* (JM109), and confirmed by DNA sequencing.

### Protein purification

#### Purification from E. coli cells

All recombinant proteins, except for pGEX6P1-GAS7b-Avitag, pGEX6P1-Nck-Avitag, and pGEX6P1-WISH-Avitag, were expressed in *E. coli* (Rosetta 2) cells cultured in LB medium containing ampicillin (0.1 mg/ml) and chloramphenicol (0.034 mg/ml). The protein expression was induced with 0.2 mM isopropyl β-d-1-thiogalactopyranoside (IPTG) when the absorbance of the culture at 600 nm [optical density of 600 nm (OD_600_)] reached 0.6 to 0.7 and then cultured overnight at 20°C and 200 rpm.

The plasmids bearing pGEX6P1-GAS7b-Avitag, pGEX6P1-Nck-Avitag, or pGEX6P1-WISH-Avitag were cotransformed with pBirAcm, which encodes biotin ligase, into *E. coli* BL21 (DE3) cells. The plasmid-containing *E. coli* cells were grown at 37°C in LB medium containing ampicillin (0.1 mg/ml). Protein expression was induced by adding 0.2 mM IPTG when the OD_600_ of the culture reached 0.6. At the same time, 0.1 mM biotin was added for biotinylation of target proteins. Then, the induced cultures were grown at 20°C for 20 hours at 200 rpm.

The *E. coli* culture was centrifuged to harvest the cells, which were stored at −80°C. The harvested *E. coli* cells from a 50-ml culture were then disrupted by sonication for 3 min in sonication buffer [10 mM tris-HCl (pH 7.5), 150 mM NaCl, 1 mM EDTA (pH 8.0), and 5% glycerol]. The cell lysate was centrifuged at 12,000*g* for 15 min at 4°C. The supernatant was incubated with 100 μl of equilibrated Glutathione Sepharose 4B beads (GE Healthcare, 17-0756-01) for 1 hour at 4°C and then centrifuged at 500*g* to collect the beads. The supernatant was removed, and the beads were washed three times with ice-cold wash buffer [10 mM tris-HCl (pH 7.5), 150 mM NaCl, and 1 mM EDTA (pH 8.0)]. PreScission Protease (GE Healthcare) was then used to cleave the GST-tag from GST-tagged proteins in ice-cold wash buffer, with rotation at 4°C overnight. The supernatant was then collected by centrifugation at 500*g* for 1 min at 4°C. All of these protein expression samples were subjected to SDS–polyacrylamide gel electrophoresis (SDS-PAGE) and stained with Coomassie Brilliant Blue (CBB). The protein concentration was measured by the intensity of each protein band based on the ImageJ software, using the marker proteins as standards or using a Qubit 4 fluorometer (Invitrogen).

#### Purification from human embryonic kidney–293 cells

The plasmids (30 μg) were transfected into human embryonic kidney–293FS cells (Thermo Fisher Scientific), at a cell density of ~8 × 10^5^ cells/ml, with 90 μl of polyethyleneimine (1 mg/ml; PEI25000, Polysciences) and 910 μl of Opti-MEM reduced serum media (Thermo Fisher Scientific) in 30 ml of FreeStyle 293 Expression Medium (Thermo Fisher Scientific, 12338026). The cells were grown at 37°C in an 8% CO_2_ atmosphere for 2 days. The cells were collected in 10-ml culture aliquots by centrifugation at 1000*g* for 5 min and then stored at −80°C. The cell pellet was suspended in 1 ml of lysis buffer [10 mM tris-HCl (pH 7.5), 150 mM NaCl, 5 mM EDTA, 0.5% Triton X-100, and 1 mM phenylmethylsulfonyl fluoride], lysed by sonication at 4°C, and then centrifuged at 12,000*g* for 15 min at 4°C. The GST fusion protein was then purified as described above. The FLAG-tagged proteins were purified by an incubation with 15 μl of anti-FLAG agarose beads (Sigma-Aldrich) at 4°C for 1 hour. The beads were washed three times with wash buffer [10 mM tris-HCl (pH 7.5), 150 mM NaCl, and 1 mM EDTA] and then incubated in 100 μl of FLAG peptide (100 μg/μl; Sigma-Aldrich) for 30 min at 4°C. The purified proteins were collected by centrifugation at 500*g* for 5 min and then analyzed by SDS-PAGE and CBB staining.

### Liposome cosedimentation assay

The final liposome concentration was 0.4 mg/ml or as described. The liposomes and proteins were mixed and incubated at 25°C for 20 min and then pelleted by centrifugation at 50,000 rpm for 20 min in a TLA100 rotor (Beckman Coulter). The pellet and supernatant were fractionated by SDS-PAGE and stained with CBB. Each protein band intensity was measured by the ImageJ software.

### Pull-down assay

To analyze protein-protein interactions, 1 μM GST-fused protein or 1 μM GST as a control was immobilized on 10 μl of Glutathione Sepharose beads (GE Healthcare) in 400 μl of the binding buffer, containing 10 mM tris-HCl (pH 7.5), 150 mM NaCl, 1 mM EDTA, and 5% (w/v) glycerol. The beads were mixed with 0.5 μM proteins for analysis, except for Nck and Grb2 (1 μM) for the interaction with N-WASP and GAS7 isoforms, at 4°C for 1 hour, followed by three washes using 1 ml of binding buffer. The proteins bound on the beads were examined by SDS-PAGE and Western blotting.

### Kinetic analysis by biolayer interferometry

The binding kinetics and affinities were measured by biolayer interferometry, using the BLItz instrument (ForteBio). Octet High Precision Streptavidin (SAX) Biosensors (Sartorius, Germany) were hydrated in the assay buffer, containing 10 mM tris-HCl (pH 7.5), 150 mM NaCl, and 1 mM EDTA, for at least 10 min. The measurement involved five steps, comprising the initial baseline, loading of biotinylated proteins, baseline equilibration, analyte association, and analyte dissociation. In this assay, biotinylated GAS7b, Nck, and WISH were each immobilized on SAX biosensor tip. First, the biosensors were soaked in the assay buffer for 30 s (initial baseline step). Then, 0.1 μM biotinylated proteins were captured on the SAX biosensors for 120 s (loading step). Next, the SAX biosensors with biotinylated proteins were washed for 30 s using 200 μl of the same buffer (baseline step). During the association step, SAX biosensors with biotinylated proteins were soaked in 0.25 μM solutions of WASP or its mutants for 120 s (association step). Last, the biosensors were soaked in the assay buffer during the dissociation step for 120 s (dissociation step). All measurements were performed in triplicate. The control values were measured using biosensors without analyte proteins of WASP or its mutants. The sensorgrams, which show the thickness of the proteins on the biosensor tip, were fit locally to a 1:1 binding model by the BLItz Pro version 1.1.0.28 software, to calculate the rate constants of association [*K*_a_ (*K*_on_)] and dissociation [*K*_d_ (*K*_off_)]. The *K*_d_ was calculated as the ratio of *K*_d_/*K*_a_ (*K*_off_/*K*_on_).

### Liposome preparation

Liposomes were prepared as described previously (*69*). Liposomes made from POPC (50475C, Sigma-Aldrich), POPE (850757C, Sigma-Aldrich), POPS (840034C, Sigma-Aldrich), DGS-NTA (790404C, Avanti), and PIP_2_ (P9763, Sigma-Aldrich). To make liposomes, lipids in organic solvents were mixed at ratios of POPC:POPE:POPS = 20:20:60, POPC:POPE:POPS = 45:45:10, or POPC:POPE:POPS:DGS-NTA:PIP_2_ = 45:45:10:10:5. The lipid mixtures were dried under nitrogen gas, followed by drying in a vacuum for 20 min. The dried lipid was swelled with 20 μl of buffer containing 10 mM tris-HCl (pH 7.5), 1 mM EDTA, and 300 mM sucrose, at 45°C for 8 min, and then 180 μl of the same buffer was added for liposome formation at 37°C for 1 hour.

### FRET measurement by spectroscopy

The time-dependent spectra were measured using a 0.1-ml cuvette in a JASCO FP-6500 spectrofluorometer with CFP or YFP proteins and their fusion proteins at a ratio of 1:6, unless indicated otherwise, in a buffer containing 10 mM tris-HCl (pH 7.5), 150 mM NaCl, and 1 mM EDTA (pH 8.0). The concentrations of other proteins are as indicated. The samples were excited at 433 nm, and emission spectra were recorded at 5-nm steps between 450 and 550 nm, with a scan speed of 2000 nm/min, at 9-s intervals. The protein complexes or liposomes were added at 45 s of the measurements.

The relative increase in the FRET efficiency was calculated using the formula for the relative proximity ratio (*E*_PR_) (*70*, *71*)

$$E_{\mathrm{PR}}={\mathrm{IA}}_{525\;\mathrm{nm}}/({\mathrm{IA}}_{525\;\mathrm{nm}}+{\mathrm{ID}}_{475\;\mathrm{nm}})$$

The IA is the emission intensity at 525 nm. The ID is the emission intensity at 475 nm. The increase in the FRET efficiency was averaged to obtain [*E*_PR_]_plt_ by the FRET values after 117 to 171 s, where the FRET reached to plateau in all our measurements here. The increase in FRET efficiency was defined as the increase from the first reading (time = 0)

$$[E_{\mathrm{PR}}\;\mathrm{increase}]={\left[E_{\mathrm{PR}}\right]}_{\mathrm{plt}}-{\left[E_{\mathrm{PR}}\right]}_{0}$$

and used for statistical comparison.

### Activation of Cdc42

Cdc42 was loaded with GTPγS, as described previously (*72*). Briefly, 0.01 mM of GTPγS or GDP was added into the buffer containing 50 mM tris-HCl (pH7.5) with 5 mM EDTA and incubated for 10 min at 30°C. After this incubation, active Cdc42 with GTPγS was mixed with the proteins at the indicated concentrations, in buffer containing 10 mM tris-HCl (pH 7.5), 1 mM EDTA, and 150 mM NaCl, at a ratio of 1:5.

### Loading of FcγRIIA on liposomes

A 0.2 μM solution of FcγRIIA cyto was phosphorylated by 0.1 μM SrcY530F protein, in a buffer containing 1 mM adenosine 5′-triphosphate (ATP), 20 mM Hepes-NaOH (pH 7.2), 10 mM MgCl_2_, 3 mM MnCl_2_, and 150 mM KCl. The reaction mixture was preincubated for 10 min at 37°C, and then Src Y530F and FcγRIIA cyto were mixed with the liposome solution at a 1:3 ratio. The liposome solution containing FcγRIIA and Src Y530F was mixed with the proteins in the buffer, containing 10 mM tris-HCl (pH 7.5), 1 mM EDTA, and 150 mM NaCl, in a 1:1 ratio.

### GUV observations by microscopy

GUVs were generated from dried lipids prepared according to the same method as for the cosedimentation assay, except for the addition of 0.5% biotin-PE [1,2-dioleoyl-*sn*-glycero-3-phosphoethanolamine-*N*-(cap biotinyl), Avanti Polar Lipids, 870273C]. The swelling was performed with 300 mM sucrose. The chamber for GUV fixation was made of two cover glasses (22 mm by 22 mm and 25 mm by 60 mm, NEO, Matsunami Glass, Japan) with double-sided tape (Nichiban, NW-15, Japan). The supported membrane was made by the absorption of the 0.2 mM dioleoylphosphatidylcholine liposomes containing 0.1% lipid biotin-PE (Avanti Polar Lipids, 870273C) that were extruded through a 100-nm pore size filter (mini extruder, Avanti Polar Lipids) in phosphate-buffered saline (PBS) for 20 min. After washing with PBS, 0.1 μM streptavidin in PBS was used to coat the supported membrane for 20 min. The streptavidin layer was then rinsed in PBS, and the GUVs containing biotin-PE were fixed. Then, the 0.05 μM His-FcγRIIA cyto was then attached to the GUVs for 15 min with 0.025 μM Src Y530F in the buffer containing ATP as above. The protein solutions were then added to the final concentrations of 1 μM GFP-GAS7b or 1 μM GFP-GAS7b, 0.5 μM mCherry N-WASP/N-WASP ΔPRR, or 0.1 μM WASP/ WASP S339Y/ WASP P359T/ WASP P373S, 0.5 μM Nck, 0.5 μM WISH, and 0.1 μM Cdc42-GTPγS. The fluorescence was observed with a confocal microscope (FV1000, Olympus) after 15 min. To analyze the protein localization on GUVs, various concentrations of GFP-GAS7 and mCherry–N-WASP were used in the presence or absence of 0.5 μM Nck, 0.5 μM WISH, and 0.1 μM Cdc42-GTPγS on GUVs containing 0.05 μM His-FcγRIIA cyto and 0.025 μM Src Y530F.

The fluorescence intensities of GFP-GAS7b, mCherry–N-WASP, and mCherry-WASP on GUVs were defined as *I* = *I*_protein on GUV_ − *I*_background_. The protein intensity on GUVs (*I*_protein on GUV_) was measured by the line of circle per GUV. The *I*_background_ is the intensity of the outer background. The GUVs displaying biased protein localization were defined as those with more than 50% fluorescence intensity changes along the circle.

### Immuno-gold staining of proteins on the monolayer membrane

The formvar membrane support of the grid for the transmission electron microscope was made with polyvinyl formvar (IA19K, Nisshin EM) and then coated with carbon using a carbon coater machine (VS100S). The mixture (0.1 mg/ml) of 45% POPC: 45% POPE: 10% POPS: 10% DGS-NTA and 5% PIP_2_ in chloroform was deposited on buffer containing 10 mM tris-HCl (pH 7.5), 100 mM NaCl, and 1 mM EDTA, at room temperature for an hour in a hole of a Teflon block for the development of the monolayered membrane, as described previously (*73*). The grid was placed onto the monolayered membrane on the Teflon block for 5 min. The proteins were then added to the grid bound with a monolayered membrane at 25°C. After 1 hour, the monolayer membrane grid with the bound proteins was rinsed with PBS and fixed with 1% glutaraldehyde for 5 min. The grid was then washed with PBS and 1% glycine in PBS for 5 min twice, to block the glutaraldehyde fixative, and then further blocked with 1% bovine serum albumin (BSA) in PBS for 5 min. After blocking, the grid was incubated with the primary antibodies [mouse anti-GAS7 (clone 2F6, TA501756, OriGene), rabbit anti–N-WASP (#4848, Cell Signaling Technology), mouse anti-WASP (#48606, Cell Signaling Technology), or rabbit anti-Cdc42 (#2466, Cell Signaling Technology)] at a 1:100 dilution for 30 min, as described in the legend to Fig. 5. Unbound antibodies were removed by four washes with 1% BSA in PBS, for 1 min each. The secondary antibodies, anti-rabbit conjugated with 5-nm gold particles (117 K1049, Sigma-Aldrich) and anti-mouse conjugated with 10-nm gold particles (038 K1022, Sigma-Aldrich) (1:100 dilution), were incubated with the grids for 30 min. Excess unbound secondary antibody was removed by four washes with 1% BSA in PBS, for 1 min each. After post-fixation with 1% glutaraldehyde for 5 min, the grids were washed with PBS for 1 min and then twice with distilled water. Last, the grids were counterstained twice with 1% uranyl acetate in distilled water, for 2 min each. The grids were dried and then observed with a transmission electron microscope (Hitachi H-7100). The distances between striations where multiple striations were observed side by side (Fig. 5H) and the area of the protein decorated on the monolayer membrane visualized in each image (Fig. 5I) were measured using ImageJ.

### Cell culture and frustrated phagocytosis

The GAS7b knockout RAW264.7 cells were cultured in Dulbecco’s modified Eagle’s medium (DMEM) supplemented with 10% fetal bovine serum and 1% penicillin-streptomycin solution at 37°C, in a humidified incubator with a 5% CO_2_ atmosphere. These cells were introduced with YFP-GAS7b-IRES-CFP-GAS7b using a retrovirus, as described previously (*9*), and were then cloned using a cell sorter. Clones with GAS7 expression similar to that of the parental cells were selected and examined for their FRET activity.

mCherry and mCherry-tagged proteins were introduced into the GAS7 knockout RAW264.7 cells expressing YFP-GAS7b-IRES-CFP-GAS7b by the retrovirus, using the vectors pMXs-mCherry, pMXs-mCherry-WISH, pMXs-mCherry-Y52AWISH, pMXs-mCherry-WASP, pMXs-mCherry-WASP S339Y, pMXs-mCherry-WASP P359T, and pMXs-mCherry-WASP P373S. After 3 days of virus transduction, the cells were subjected to the frustrated phagocytosis assay.

For frustrated phagocytosis, glass-bottom dishes (IWAKI) were coated with human IgG (1 mg/ml) for 40 min at room temperature. The excess antibodies were removed by three rinses with DMEM. The cells were treated with 0.1% DMSO, 10 μM ML141, and 10 μM PP2 in 500 μl of cell suspension, for 20 min before seeding. The cells were then seeded and placed in an incubator at 37°C with a 5% CO_2_ atmosphere, for 20 min. Afterward, the cells were fixed with 3% paraformaldehyde for 20 min. The fixed samples were reduced with PBS containing 0.1% NaBH_4_ for 5 min. The samples were then stored in PBS with 1% polyvinyl alcohol and 10 mM cysteamine before observations with a TCS SP8 microscope (Leica) for FLIM.

### FLIM analysis

FLIM measurements were performed using a Leica TCS SP8 confocal microscope (Leica Microsystems) equipped with a FLIM 433 WLL (PicoQuant) imager and an argon laser. Images were acquired using a 63× water-immersion objective lens. The CFP was excited with a pulsed laser source at 440 nm, and the lifetime of CFP was measured in the mCherry or mCherry-tagged protein-expressing cells. The lifetime was calculated by mean lifetime τ (nanseconds) fitting in the LASX software (Leica). The pseudocolor of the lifetime was generated by the royal lookup table (LUT) in ImageJ.

### Western blotting and antibodies

After SDS-PAGE, the proteins in the gel were transferred onto a membrane (Immobilon P, IPVH00010, Merck Millipore) using a Trans-Blot SD Semi-Dry Transfer Cell (Bio-Rad). The membrane was blocked with 5% skim milk in PBS, supplemented with 0.05% Tween 20 (PBS-T). The membranes were incubated with the primary antibody: mouse anti-GAS7 (clone 2F6, TA501756, OriGene), anti-Grb2 (Sc-8034, OriGene), anti-Nck (SC-20026, Santa Cruz Biotechnology), anti–N-WASP (#4848, Cell Signaling Technology), anti-WASP (#48606, Cell Signaling Technology), anti-SPIN90 (Ab88467, Abcam), anti-mCherry (71615, Cell Signaling Technology), and anti–glyceraldehyde-3-phosphate dehydrogenase (SC16657, Santa Cruz Biotechnology) at a 1:10,000 dilution, followed by an anti-mouse or anti-rabbit IgG alkaline phosphatase conjugate (Promega) secondary antibody in PBS-T at a 1:10,000 dilution. The alkaline phosphatase was detected by 5-bromo-4-chloro-3-indolyl phosphate/nitro blue tetrazolium (Roche).

### Statistical analyses

All data are means ± SE or mean ± SD, as indicated in the legends. Data for each condition were obtained from at least three independent biological replicated experiments. The data that had a normal distribution were subjected to the analysis of variance (ANOVA) test, followed by the Tukey and Dunnett tests, for comparisons among all the means or between each mean and that of the control. The data that did not have normal distribution were analyzed by the Kruskal-Wallis test, followed by Dunn’s test. These statistical analyses were performed using GraphPad Prism 8. A value of *P* < 0.05 was considered significant.

## Notes

View/request a protocol for this paper from *Bio-protocol*.
