# Supplementary material for: Small GTPase Cdc42, WASP, and scaffold proteins for higher-order assembly of the F-BAR domain protein
Source: Sci Adv. 2023 Apr 26;9(17):eadf5143. doi: 10.1126/sciadv.adf5143 (PMC10132759; doi:10.1126/sciadv.adf5143)

Science Advances
AAAS

Supplementary Materials for

Small GTPase Cdc42, WASP, and scaffold proteins for higher-order assembly of the F-BAR domain protein

Wan Nurul Izzati Wan Mohamad Noor *et al.*

Corresponding author: Shiro Suetsugu, suetsugu@bs.naist.jp

Sci. Adv. **9**, eadf5143 (2023)
DOI: 10.1126/sciadv.adf5143

This PDF file includes:

Figs. S1 to S5

Figure S1. Binding of tagged GAS7b and non-tagged GAS7b to liposomes

(A) Representative images of SDS-PAGE gels after purification of the proteins used in this study.

(B) Binding curves of GAS7b and GFP-GAS7b (1 μM) to liposomes containing 20% POPC, 20% POPE, and 60% POPS by a liposome co-sedimentation assay. The POPS concentrations ranged from 0 to 630 μM. The exponential binding curves fitted for each set of data points are shown. The means ± SE are shown (n=3).

(C) Fluorescent emission of FRET donor (CFP-GAS7b) and acceptor (YFP-GAS7b) mixture before and after the addition of liposomes (0.25 mg/ml).

Figure S2. Protein-protein interactions of GAS7, N-WASP/WASP, Nck, and WISH

(A) Binding of N-WASP to GAS7 splicing variants. N-WASP (1 μM) was immobilized on the beads by glutathione as a GST fusion protein, and then incubated with GAS7cb, GAS7b, or GAS7d (1 μM). After washing, the bound proteins were analyzed by SDS-PAGE followed by western blotting using the anti-GAS7 antibody, as shown in the upper panel. The protein inputs before washing are shown in the lower panel after SDS-PAGE and CBB staining. GST was used as a negative control.

(B) The protein inputs before washing are shown after SDS-PAGE and CBB staining for Figure 2B

(C) The dissociation constant (K_D), association (k_a), and dissociation (k_d) rate constants values were measured by fitting a locally binding curve, related to Figures 2D, 2G, and 2J.

(D-E) The protein inputs before washing are shown after SDS-PAGE and CBB staining for Figure 2E and 2F, respectively.

(F) Binding of N-WASP, WISH, and Nck (0.5 μM) to GAS7b and its ΔFFL2 and K208A/K209A mutants (1 μM), as in (A).

(G-H) The protein inputs before washing are shown after SDS-PAGE and CBB staining for Figure 2H and 2I, respectively.

Figure S3. Quantification of increased FRET of GAS7

Increase in the GAS7 FRET efficiency (E_{PR}). The protein solution contained CFP-GAS7b + YFP-GAS7b in the presence of 0.5 μM N-WASP, 0.5 μM Nck, 0.5 μM WISH, and 0.1 μM FLAG-tagged Cdc42, purified from HEK293 cells, as well as 0.1 μM His-tagged Cdc42 that was purified from *E. coli*, loaded with GTPγS or GDP. Liposomes were composed of 0.2 mg/ml 45% POPC, 45% POPE, and 10% POPS. The means ± SD are shown (n=3).

The P-values were obtained using the one-way ANOVA with Tukey's post hoc analysis. Significance values is **p<0.01.

Figure S4. Protein localization on the GUVs in the presence and absence of FcγRIIA cyto and Src Y530F

(A) Representative images of GUVs (0.1 mM lipids containing 45% POPC, 45% POPE, 10% POPS, 10% DGS-NTA, 5% PIP_2, and 0.5% biotin-PE) in the presence or absence of 0.05 μM His-FcγRIIA cyto and 0.025 μM Src Y530F by differential interference contrast (DIC) and their associated proteins by the fluorescence of GFP and mCherry. The proteins were 1 μM GFP-GAS7b, 0.5 μM mCherry-N-WASP, 0.5 μM Nck, 0.5 μM WISH, 0.1 μM Cdc42(GTPγS), 0.05 μM His-FcγRIIA cyto, and 0.025 μM Src Y530F, as indicated in the figure. Scale bars: 5 μm.

(B) Frequency of GUVs with the biased localization of GFP-GAS7b in (A). The means ± SE are shown (n=3)

The P-values were obtained by the one-way ANOVA with Dunnett's post hoc analysis. Significant values are ***$p<0.001$ and ****$p<0.0001$.

Figure S5. Striations of the protein assembly with GAS7b on the membrane

(**A**) Representative transmission electron microscopic images of monolayered membranes incubated with 0.1 μM GAS7b, 0.1 μM N-WASP, 0.1 μM Nck, 0.1 μM WISH, and 0.1 μM Cdc42(GTPγS).

(**B**) Representative transmission electron microscopic images of monolayered membranes incubated with 0.1 μM GAS7b, 0.1 μM N-WASP, 0.1 μM Nck, 0.1 μM WISH, and 0.1 μM Cdc42(GTPγS), 0.05 μM His-FcγRIIA cyto, and 0.025 μM Src Y530F.

Scale bars=100 nm.

Fig. S1

A

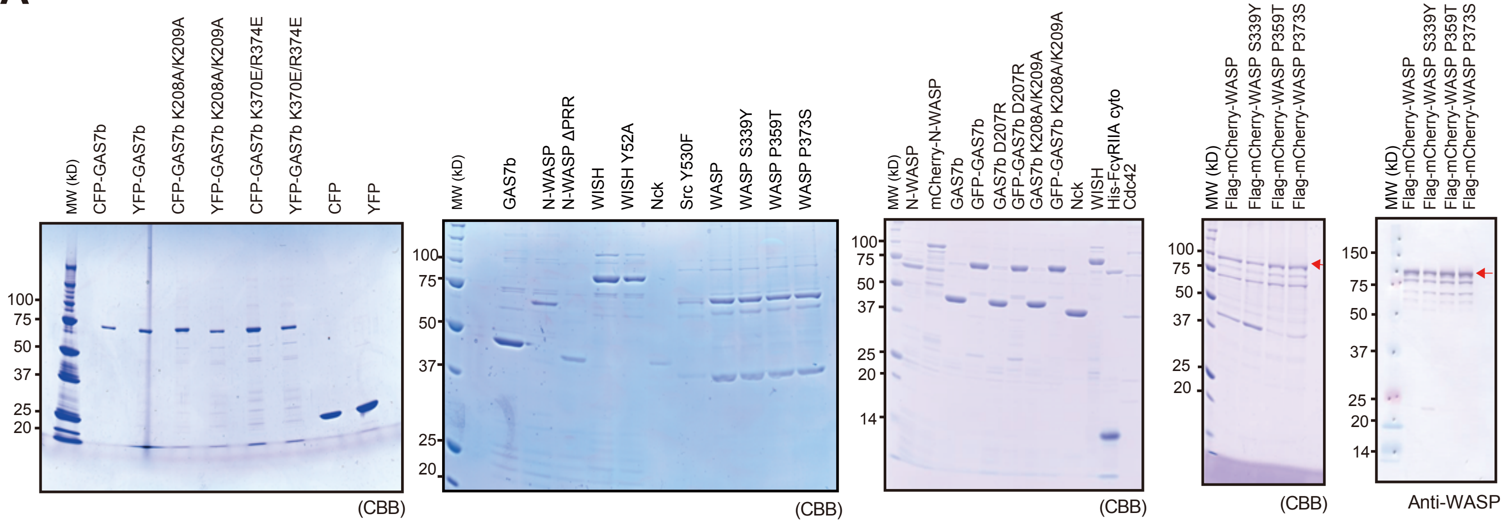

B

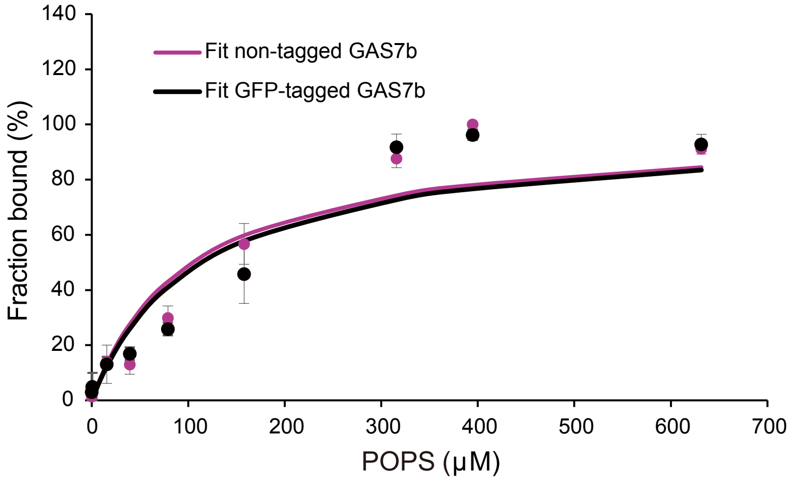

	non-tagged GAS7b	GFP-tagged GAS7b
K_D (μM POPS)	100.86	110.02
Bmax	98	98
R^2	0.97	0.96

C

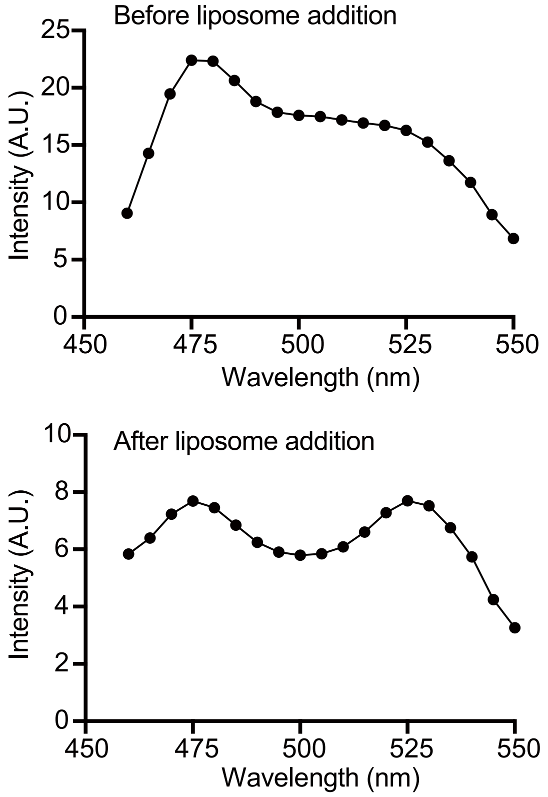

Fig. S2

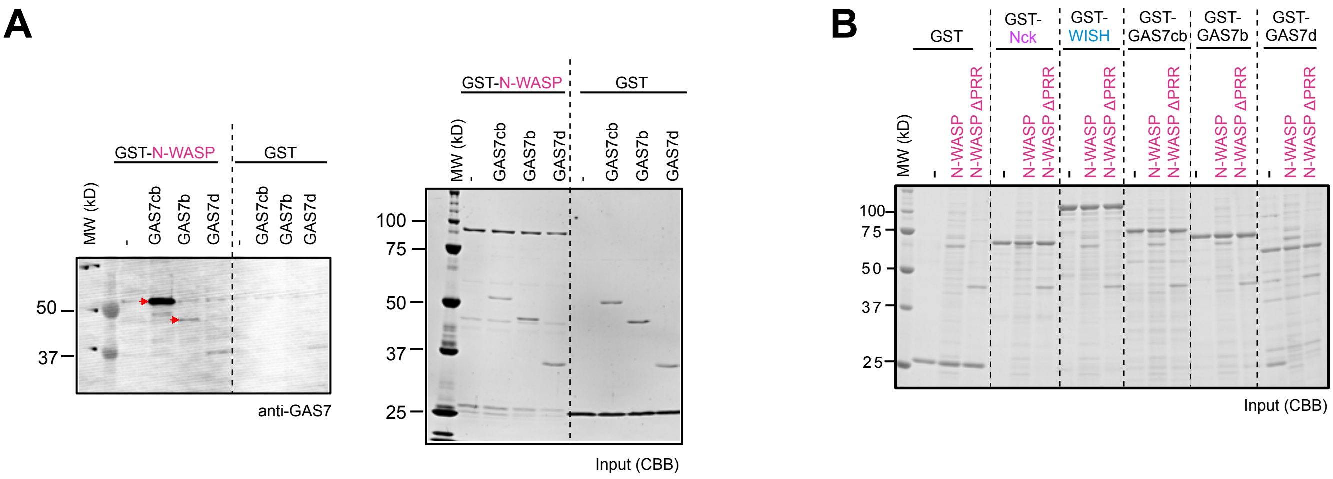

C

	GAS7b sensor				Nck sensor				WISH sensor			
	K_D (nM)	k_a (1x10⁴ /Ms)	k_d (1x10⁻⁴/s)	R^2	K_D (nM)	k_a (1x10⁴ /Ms)	k_d (1x10⁻⁴/s)	R^2	K_D (nM)	k_a (1x10⁴ /Ms)	k_d (1x10⁻⁴/s)	R^2
WASP	4.11 ± 0.30	19.58 ± 0.98	8.11 ± 0.88	0.99	2.40 ± 0.48	5.67 ± 0.11	1.35 ± 0.25	0.99	4.55 ± 0.79	6.88 ± 0.66	2.99 ± 0.24	0.99
WASP S339Y	7.13 ± 0.24	18.03 ± 1.10	11.33 ± 0.84	0.99	3.03 ± 0.20	4.80 ± 0.16	1.45 ± 0.07	0.99	7.71 ± 0.69	8.92 ± 1.19	6.65 ± 0.47	0.99
WASP P359T	6.89 ± 0.12	15.40 ± 0.66	10.64 ± 0.65	0.99	6.32 ± 0.96	9.94 ± 1.41	5.89 ± 0.04	0.99	14.62 ± 1.88	6.86 ± 1.76	9.74 ± 2.67	0.99
WASP P373S	14.98 ± 0.98	11.26 ± 0.34	16.93 ± 1.41	0.99	4.78 ± 0.58	6.68 ± 0.31	3.24 ± 0.53	0.99	13.28 ± 2.24	6.39 ± 0.74	8.35 ± 1.32	0.99

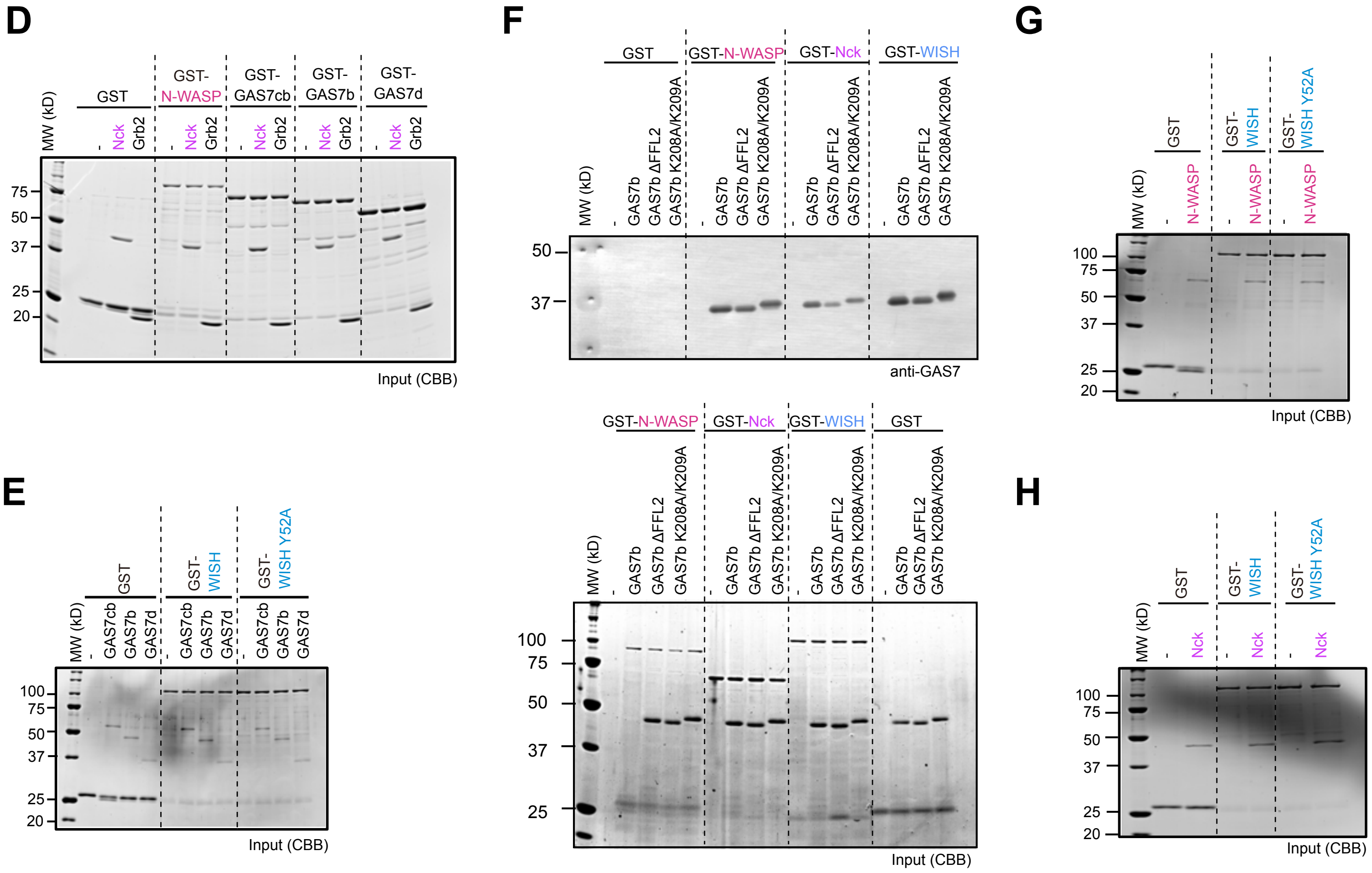

Fig. S3

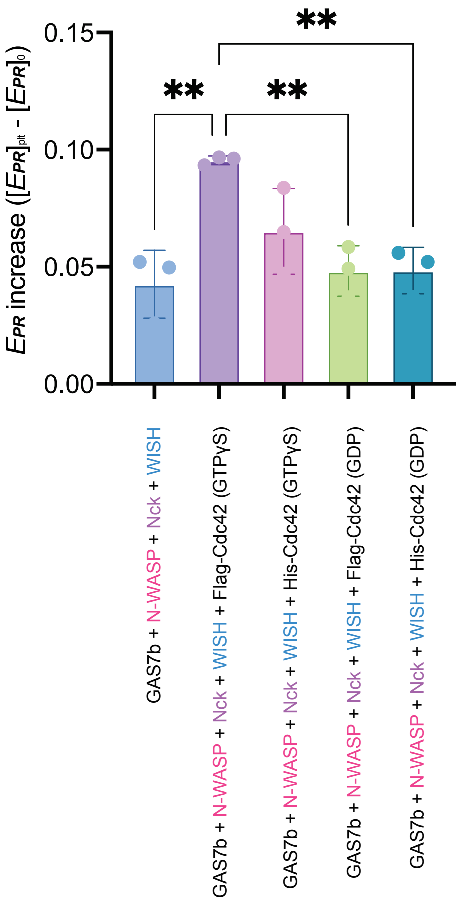

E_PR increase ([E_PR]_plt - [E_PR]_0)
0.15
0.10
0.05
0.00
**
**
**
GAS7b + N-WASP + Nck + WISH
GAS7b + N-WASP + Nck + WISH + Flag-Cdc42 (GTPγS)
GAS7b + N-WASP + Nck + WISH + His-Cdc42 (GTPγS)
GAS7b + N-WASP + Nck + WISH + Flag-Cdc42 (GDP)
GAS7b + N-WASP + Nck + WISH + His-Cdc42 (GDP)

Fig. S4

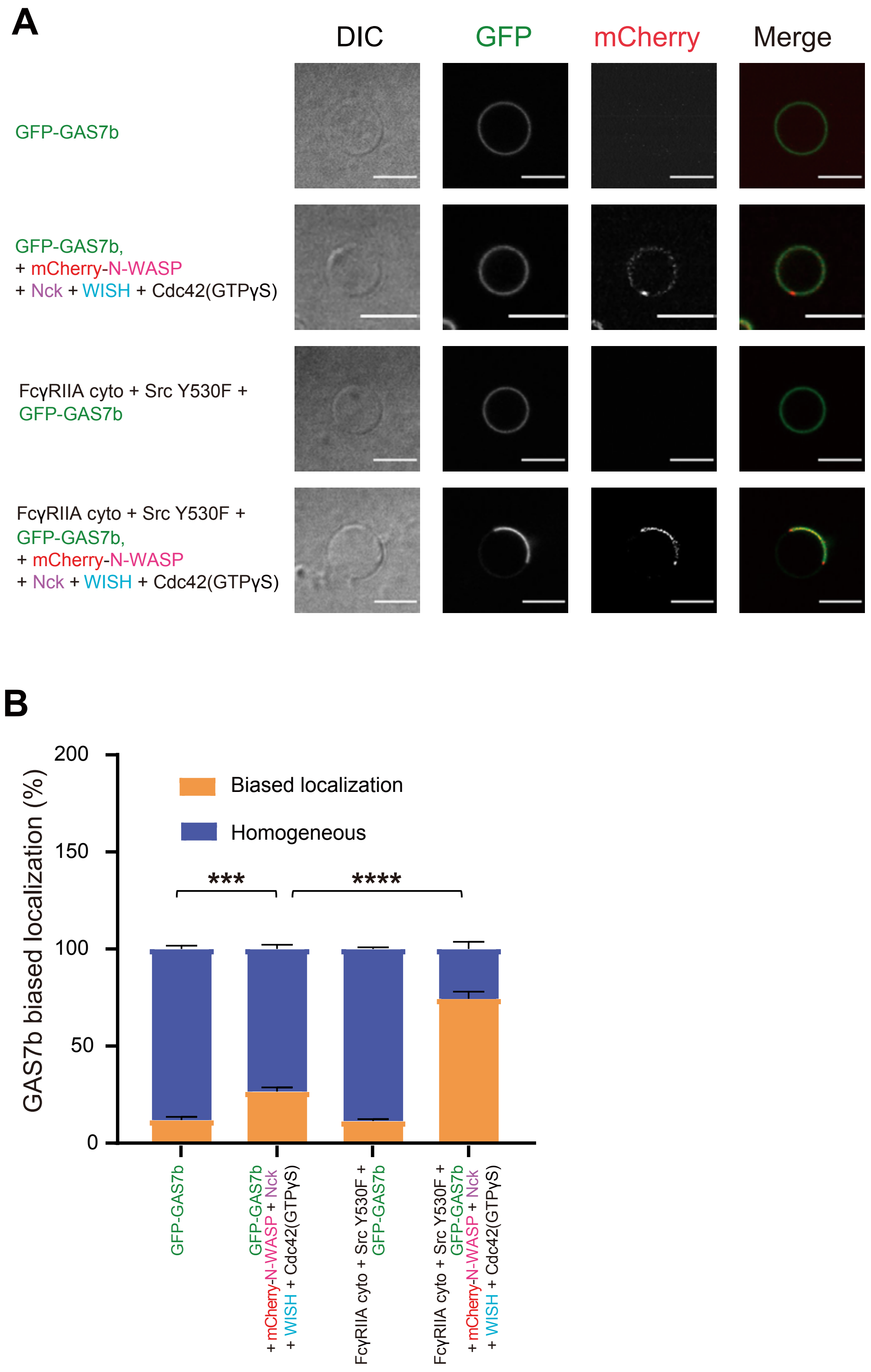
A
DIC
GFP
mCherry
Merge
GFP-GAS7b
GFP-GAS7b,
+ mCherry-N-WASP
+ Nck + WISH + Cdc42(GTPγS)
FcγRIIA cyto + Src Y530F +
GFP-GAS7b
FcγRIIA cyto + Src Y530F +
GFP-GAS7b,
+ mCherry-N-WASP
+ Nck + WISH + Cdc42(GTPγS)
B
GAS7b biased localization (%)
200
150
100
50
0
Biased localization
Homogeneous
***
****
GFP-GAS7b
GFP-GAS7b
+ mCherry-N-WASP + Nck
+ WISH + Cdc42(GTPγS)
FcγRIIA cyto + Src Y530F +
GFP-GAS7b
FcγRIIA cyto + Src Y530F +
GFP-GAS7b
+ mCherry-N-WASP + Nck
+ WISH + Cdc42(GTPγS)

Fig. S5

A

GAS7b + Cdc42 (GTPγS) +
N-WASP + Nck + WISH

B

GAS7b + Cdc42 (GTPγS) +
N-WASP + Nck + WISH +
FcγRIIA cyto + Src Y530F

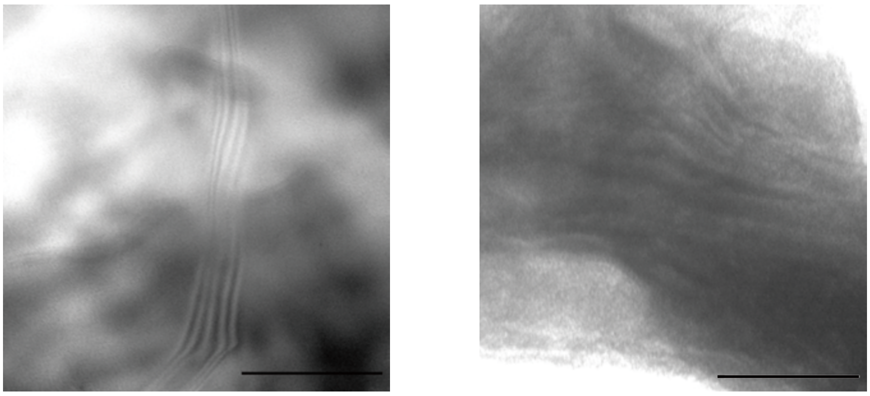

Supplement: Supplementary file 1 — Figs. S1 to S5 [file sciadv.adf5143_sm.pdf]
